# Continuous and efficient elastocaloric air cooling by coil-bending

**DOI:** 10.1038/s41467-023-43611-6

**Published:** 2023-12-02

**Authors:** Xueshi Li, Peng Hua, Qingping Sun

**Affiliations:** 1 Department of Mechanical and Aerospace Engineering, The Hong Kong University of Science and Technology, Kowloon, Hong Kong, China; 2 HKUST Shenzhen-Hong Kong Collaborative Innovation Research Institute, Futian, Shenzhen, Guangdong, China

**Keywords:** Energy science and technology, Mechanical engineering, Applied physics

## Abstract

Elastocaloric cooling has emerged as an eco-friendly technology capable of eliminating greenhouse-gas refrigerants. However, its development is limited by the large driving force and low efficiency in uniaxial loading modes. Here, we present a low-force and energy-efficient elastocaloric air cooling approach based on coil-bending of NiTi ribbons/wires. Our air cooler achieves continuous cold outlet air with a temperature drop of 10.6 K and a specific cooling power of 2.5 W g^−1^ at a low specific driving force of 26 N g^−1^. Notably, the cooler shows a system coefficient of performance of 3.7 (ratio of cooling power to rotational mechanical power). These values are realized by the large specific heat transfer area (12.6 cm^2^ g^−1^) and the constant cold zone of NiTi wires. Our coil-bending system exhibits a competitive performance among caloric air coolers.

## Abstract (web summary)

In this work, authors demonstrate a low-force and energy-efficient elastocaloric air cooling approach based on coil-bending of NiTi ribbons/wires. The device achieves continuous cold outlet airflow with a temperature drop of 10.6 K and a specific cooling power of 2.5 W g^−1^ at a low specific driving force of 26 N g^−1^.

## Introduction

It is estimated that there are 1.6 billion air conditioners used for space cooling worldwide^1^, and most of these are based on conventional vapor-compression (VC) technology. The leaked VC refrigerants (e.g., chlorofluorocarbons and hydrochlorofluorocarbons) account for 7.8% of all greenhouse-gas emissions^1–4^, which are rapidly increasing due to the expanding demands of cooling. Compared with VC, the fast-developing solid-state elastocaloric cooling^2–4^ based on stress-induced phase transformation^5–12^ has demonstrated advantages in eco-friendly refrigerants, cooling capacity, and energy efficiency^12^. NiTi shape memory alloy (SMA)^13–15^ has been widely used in elastocaloric cooling prototypes due to its super-long fatigue life^16–20^ and large entropy change^21,22^. The binary NiTi SMA with full phase transition exhibits a remarkable entropy change up to 0.32 J cm^−3^ K^−1^ under a maximum tensile stress of 750 MPa and 0.36 J cm^−3^ K^−1^ under a maximum compressive stress of 950 MPa^22^. These values notably surpass the entropy change exhibited by hydrofluorocarbon-32 refrigerants (0.035 J cm^−3^ K^−1^) in VC refrigeration^23^. Over the past decade, there have been substantial advancement in the development of SMA-based elastocaloric cooling prototypes. These prototypes have utilized various driving modes and refrigerant structures, including compression-based thin-walled tubes^24–32^, topology-designed tubular structures^33–35^, tension-based wires^23,36–39^ and sheets^40–44^, bending-based wires^45–47^ and sheets^48,49^, and torsion-based wires^50^ (Supplementary Note S10 and Table S6). Commercial air conditioning systems require compact size, high safety, and high energy efficiency. However, achieving a cooling power of one kilowatt in a NiTi-based elastocaloric cooling prototype demands a massive linear or hydraulic actuator with a minimum driving force of 100 kN (equivalent to 260 N g^−1^ or 900 MPa compressive stress)^35^. The use of large actuators not only occupies excessive space but also poses potential safety risks, making them unsuitable for residential applications. Furthermore, the efficiency of an actuator decreases with its size and driving force^51–53^. The inclusion of additional transmission mechanisms and mechanical components in linear actuators introduces substantial friction losses and reduces energy efficiency. Consequently, the large driving force^51,52^ and low energy efficiency of the uniaxial loading^5,53^ have emerged as notable barriers to the commercialization of elastocaloric air conditioning.

Bending is a promising loading mode in reducing the driving force and enhancing the energy efficiency^45,49^. The specific driving force to bend a 0.5 mm-thick NiTi sheet is remarkably low, measuring only 5.64 N g^−1^ (with maximum tensile and compressive stresses of 550 MPa and 800 MPa, respectively)^49^. The value is more than two orders of magnitude lower than the tension-specific driving force (315 N g^−1^, equivalent to a tensile stress of 500 MPa)^36^ and compression-specific driving force (260 N g^−1^, equivalent to a compressive stress of 900 MPa)^35^ of the NiTi SMA. Benefiting from the low driving force, the elastocaloric cooler utilizing bending actuation offers the advantage of a small volume, making it a promising candidate for cooling electronic circuits^46^. More importantly, rotary motors can be directly used to bend NiTi ribbons/wires, which avoids the energy loss in motion transmission and transformation. Mechanical efficiency, i.e., the ratio of output mechanical power to input electrical power, quantifies the energy efficiency of motors. The mechanical efficiency (85%) and power density (1.0 MW m^−3^) of rotary motors are much higher than that of hydraulic actuators (50%) and linear motors (0.004 MW m^−3^)^5,51–54^. It is therefore more desirable to use rotary motors in elastocaloric air coolers for an enhancement in the system coefficient of performance (COP), which is the ratio of cooling power to input power^45,52^. Additionally, direct and continuous air cooling not only minimizes heat loss between heat transfer processes, but also eliminates the need for additional heat exchangers and avoids potential liquid sealing issues^14,37,55^. However, a clear technological route and a compact system design are required to achieve continuous and energy-efficient elastocaloric air cooling through the bending of NiTi SMA^45,49^.

In this work, we report an alternative actuation named coil-bending that activates phase transformation of NiTi at a low driving force. We build a compact elastocaloric air cooler based on the coil-bending of NiTi wires, where the coiled wires form a hot zone, and the uncoiled wires form a constant cold zone. Cold air can be continuously produced from the elastocaloric air cooler by pumping the air through the constant cold zone. Serial and parallel air channels are used to obtain high-temperature drop of the outlet air and high system COP, respectively. Our coil-bending actuation not only reduces the driving force, but also substantially enhances the energy efficiency of elastocaloric air coolers.

## Results

### Principle of coil-bending actuation

**Fig. 1:**
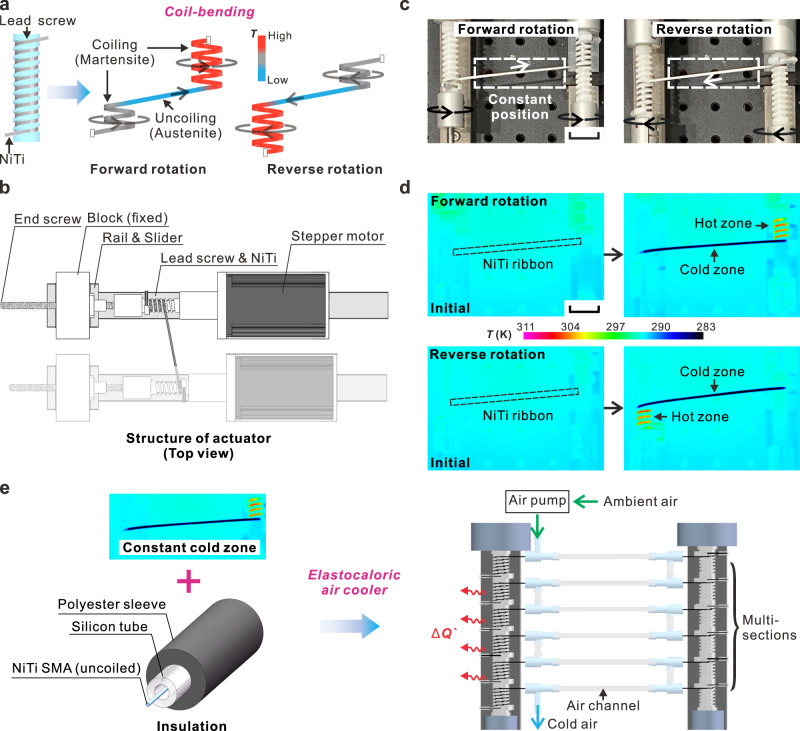
Principle of our elastocaloric air cooler by coil-bending of NiTi wires/ribbons. **a** Schematic drawing of coil-bending. **b** Top view of the coil-bending system of NiTi SMA. **c** Photos of a NiTi ribbon maintaining the same position during operation. Scale bar, 25 mm. **d** Thermographic images of the NiTi ribbon subjected to adiabatic coil-bending. Scale bar, 25 mm. **e** Insulation and assembly of multiple NiTi samples into a cooling array.

Our coil-bending principle and system design are schematically shown in Fig. 1a. A demonstration of our coil-bending elastocaloric air cooler is recorded in the Supplementary Movie 1. NiTi SMA wires/ribbons were coiled into the grooves of a lead screw, which resulted in their bending-induced martensitic transformation with a release of latent heat. During the uncoiling, the NiTi SMA sample reversely transformed into its initial straight shape in austenite phase, absorbing heat from the ambient environment. Figure 1b shows the coil-bending actuator. Both ends of the NiTi SMA sample were initially coiled onto two independent lead screws, which were driven independently by two stepper motors. As the two motors rotated in the same direction, the NiTi SMA sample was uncoiled and coiled from one lead screw to the other. Reciprocal coiling/uncoiling was achieved by cyclically changing the rotating direction (forward and reverse). Despite the motion of coiling/uncoiling, the uncoiled part of the NiTi SMA sample remained at the constant position (Fig. 1c). This was realized by designing the lead screws and motors to move linearly along the rails, compensating for the displacement arising from the rotation of the helical lead screws. A thermographic photo (Fig. 1d) shows that the coiled part of the NiTi ribbon formed a hot zone while the uncoiled part of NiTi ribbon formed a constant cold zone. Based on this principle, we constructed an elastocaloric coil-bending air cooler (Fig. 1e) using an array of six serially connected NiTi SMA samples, where the uncoiled samples were insulated in silicon tubes. Polyester sleeves were used to further enhance the heat insulation. Ambient air was pumped through and cooled by the uncoiled NiTi samples in the constant cold zone. The released heat of the hot coiled NiTi ribbon/wire is transferred to the lead screw through conduction and to the surrounding still air through natural convection. The characteristic heat transfer time (*t*_h_) of the NiTi wire in contact with the steel lead screw was 0.61 s, which is much lower than the smallest half cycle (1.75 s at the frequency of 0.286 Hz). Thus, the heat of the hot coiled NiTi sample is mostly transferred to the lead screw through conduction and the ambient environment through natural convection (see Supplementary Note S5). The temperature of the lead screw slightly increased due to heat accumulation and eventually stabilized, which is further discussed in Supplementary Note S7.

### Elastocaloric effect of NiTi ribbons/wires subjected to coil-bending

**Fig. 2:**
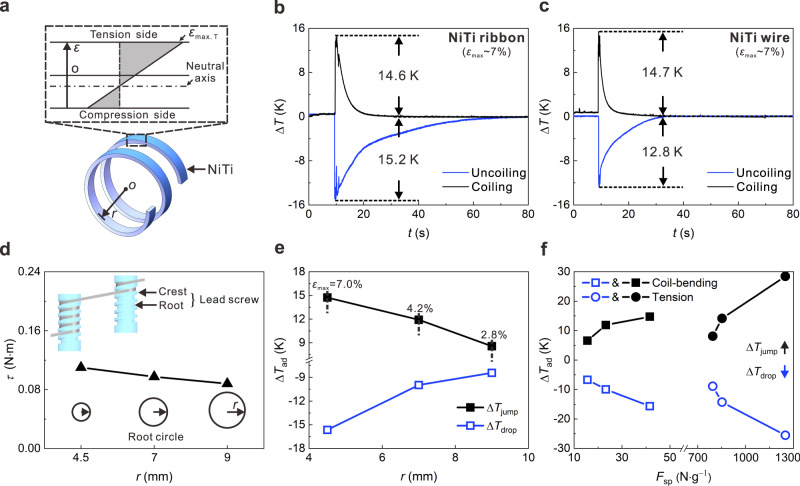
Characterization of the elastocaloric effect of NiTi ribbons and wires subjected to coil-bending. **a** Strain distribution along the thickness of a coil-bent NiTi SMA. **b** Adiabatic temperature changes of a 0.5-mm-thick NiTi ribbon during fast coiling and uncoiling. **c** Adiabatic temperature changes of a 0.5-mm-diameter NiTi wire during fast coiling and uncoiling. **d** Required torques for the coil-bending of the NiTi ribbon for different root radiuses of the lead screw. **e** Adiabatic temperature jumps and drops of the NiTi ribbon versus the root radius of the lead screw. **f** Adiabatic temperature jumps and drops of the NiTi ribbon versus the specific driving force under coil-bending compared with that of tension. Source data are provided as a Source Data file.

The elastocaloric effect of a NiTi ribbon/wire under coil-bending depends on the radius of the root circle of the lead screw (*r*, see Supplementary Note S1). In this study, we employed three lead screws with different r values (4.5 mm, 7 mm, and 9 mm), resulting in different maximum surface tensile strains in the samples^45,49^ (*ε*_max_ = 7.01%, 4.28%, and 2.88%, respectively. See “Methods” section and Supplementary information). Figure 2b presents the adiabatic temperature changes (∆*T*_ad_) of a 0.5-mm-thick NiTi ribbon during fast coiling (0.5 s) and uncoiling (0.5 s) using the lead screw with an *r* value of 4.5 mm. The results showed that the adiabatic temperature jump (∆*T*_jump_) and temperature drop (∆*T*_drop_) of the NiTi ribbon were 14.6 K and 15.2 K, respectively. It should be noted that the measured ∆*T*_drop_ of the NiTi ribbon is larger than the ∆*T*_jump_, due to conduction heat losses between the coiled NiTi ribbon and the lead screw. The thermal conduction rate is dependent on the conductivity of materials, contact area, and the temperature difference (Supplementary Note S6). Figure 2c shows the ∆*T*_ad_ of a 0.5 mm-diameter NiTi wire used in our air cooler with the same lead screw (*r* = 4.5 mm), where the ∆*T*_jump_ and ∆*T*_drop_ were 14.7 K and 12.8 K, respectively.

We also investigated the effect of *r* on the required torque (*τ*) and the resulting ∆*T*_ad_ of the NiTi ribbon. The applied *τ* on the lead screw in the forward/reverse rotation was measured by a torque sensor (see Supplementary information), where the torques in the forward and the reverse rotations are the same. The *τ* increased by 25% (from 0.088 N m to 0.11 N m), as the *r* decreased from 9 mm to 4.5 mm (Fig. 2d). Figure 2e shows the corresponding ∆*T*_ad_ of the NiTi ribbon under coil-bending with different *r* values. The ∆*T*_ad_ increased monotonously from 9 K to 16 K as the *r* decreased. Figure 2f shows that the specific driving force *F*_sp_ (41 N g^−1^) of coil-bending is more than an order of magnitude lower than that of tension (800 N g^−1^) using the same material. The coil-bending achieved a comparable ∆*T*_ad_ (up to 16 K under 41 N g^−1^) to that of the tension (16 K under 800 N g^−1^)^38^. The ∆*T*_ad_ is sufficient for household air conditioning (suitable room temperature is between 293–299 K)^56^. More importantly, the low *F*_sp_ of the coil-bending permits the use of small and quiet actuators for residential applications.

### Performance of the elastocaloric air cooler

To improve the convection heat transfer efficiency^34^, NiTi wires with a diameter of 0.5 mm and a large specific heat transfer area *A*_spht_ of 12.6 cm^2^ g^−1^ were used in our coil-bending elastocaloric air cooler. The *F*_sp_ of the NiTi wires under coil-bending was 26 N g^−1^, which is only two-thirds of that of the NiTi ribbons. We developed serial and parallel configurations for the air channels. The serial connection of the air channels enables a pre-cooling of the inlet airflow of the subsequent tube sections by the cold uncoiled NiTi wires from the previous tube sections, leading to a large temperature drop in the airflow. In contrast, the airflow is only cooled by a single uncoiled NiTi wire in the parallel configuration, which results in a large temperature difference and a high heat transfer rate between the airflow and the NiTi wire. A high specific cooling power (SCP) is achieved by the parallel configuration. The thermodynamic cycles are shown in Supplementary Note S6.

**Fig. 3:**
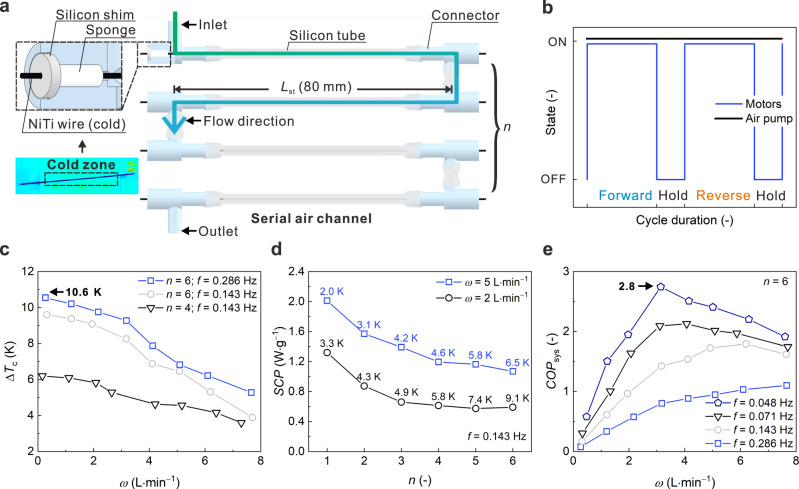
Assembly and cooling performances of our elastocaloric air cooler with serial air channels. **a** Configuration of the serial air channels. **b** Operating sequences of the motors and the air pump. **c** Δ*T*_c_ of the outlet air with different number of sections (*n*) of the air channels versus the flow rate (*ω*). **d** SCP of the elastocaloric air cooler with different *n*, where the corresponding Δ*T*_c_ is shown near each mark. **e** COP_sys_ of the elastocaloric air cooler versus *ω* at different operating frequencies (*f*). Source data are provided as a Source Data file.

**Fig. 4:**
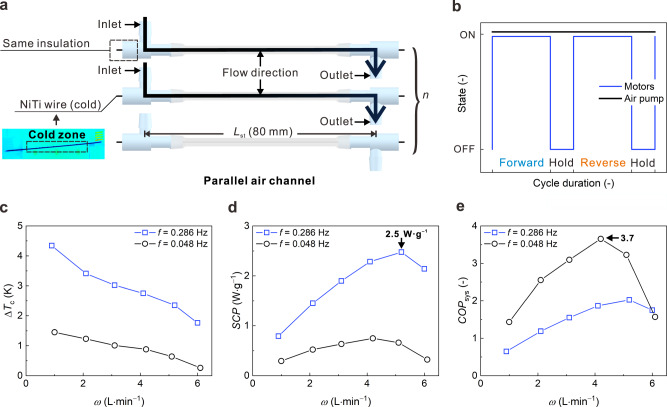
Assembly and cooling performances of our elastocaloric air cooler with parallel air channels. **a** Configuration of the parallel air channels. **b** Operating sequences of the motors and the air pump. **c** Δ*T*_c_ of the outlet air at different *f* versus *ω*. **d** SCP of the elastocaloric air cooler at different *f* versus *ω*. **e** COP_sys_ of the elastocaloric air cooler at different *f* versus *ω*. Source data are provided as a Source Data file.

As depicted in Fig. 3a, the serially connected air channels consisted of multiple sections to increase the heat transfer area and time. The uncoiled part of each NiTi wire was inserted into a silicon tube, with both ends sealed by silicon shims and sponges to prevent air leakage. The effective length of each NiTi wire in the silicon tube for heat transfer was 80 mm. Airflow was pumped into the silicon tubes and cooled by the uncoiled NiTi wires. By increasing the number of tube sections (*n*, shown in Figs. 3a and 4a), the total length of NiTi wires in the air channel (*L*_ht_) was extended to 80*n* mm. Figures 3b and 4b show the schematic operating principle of our air cooler in one elastocaloric cooling cycle, which is comprised of four stages: forward motor rotation, holding for heat transfer between the airflow and NiTi wires, reverse motor rotation, and a second holding for the heat transfer. The motors operated intermittently to save energy, and the air pump worked continuously during the operation. Increasing the *L*_ht_ and operating frequency (*f*) resulted in a cooler outlet air at a given flow rate (*ω*), as shown in Fig. 3c. The temperatures of the inlet airflow and the outlet airflow were measured by thermocouples, and the average temperature drop of the outlet airflow in the steady state was determined as Δ*T*_c_. It should be noted that the temperature of the outlet airflow has fluctuations, which is further discussed in Supplementary Note S3. The Δ*T*_c_ decreased monotonically as the *ω* increased in Fig. 3c. The Δ*T*_c_ under the configuration of *n* = 6 and *f* = 0.286 Hz reached 10.6 K. Although a larger Δ*T*_c_ can be obtained by increasing the *L*_ht_, the smaller temperature difference between the airflow and NiTi wires will lead to lower heat transfer efficiency. At the given *ω* and *f*, the SCP of the air cooler decreased with the *n*, while the corresponding Δ*T*_c_ increased with the *n* (Fig. 3d). The decrease in SCP was undesirable, as it led to a decrease in the system COP (Fig. 3e), which was calculated as the ratio of the cooling capacity to the outputted mechanical power of the rotary motors (Supplementary information). Although the SCP increased with the *f*, the COP of the air cooler decreased with the *f* due to the increased outputted mechanical power of the motors^28,40^. Figure 3e shows that a maximum system COP can be obtained at an optimum airflow rate (*ω*), depending on the *f*. At the *f* of 0.048 Hz, the air cooler with six serially connected air channels achieved a system COP of 2.8. Reducing the operating frequency led to a slower motion of the cold NiTi wires and a longer contact time for sufficient heat transfer between the air and the NiTi wires.

In contrast to the serial air channels, air in the parallel connection was only cooled by a single NiTi wire before being outputted (Fig. 4a). The large temperature difference (nearly 10.2–14.7 K) between the air and the NiTi wire enabled a high convective heat transfer rate. Although the Δ*T*_c_ was reduced in the parallel air channel design due to the short heat transfer time (Fig. 4c), the SCP was notably enhanced to 2.5 W g^−1^ by the increased heat transfer rate (Fig. 4d). Benefiting from the improved SCP of the parallel air channels, the elastocaloric air cooler achieved a high system COP of 3.7 at the operating conditions of *ω* = 4.3 L min^−1^ and *f* = 0.048 Hz.

**Fig. 5:**
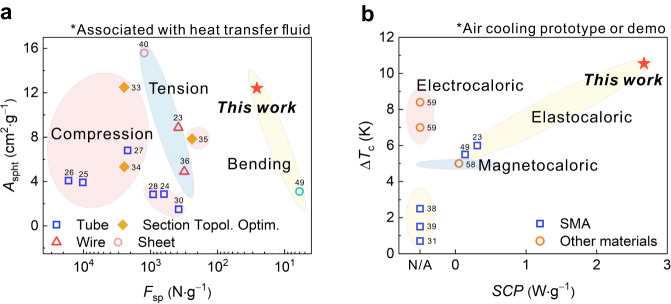
Comparison of cooling performance. **a** Comparison of the specific heat transfer area (*A*_spht_) and the specific driving force (*F*_sp_) of this work with those of previous NiTi elastocaloric prototypes. **b** Comparison of the cooling performances of this work with reported caloric air-cooling prototypes and demos (SCP values at the temperature span of 0 K). Source data are provided as a Source Data file.

With the same *F*_sp_ and *A*_spht_ of the coil-bent NiTi wires, the serial connection achieved a high Δ*T*_c_ of 10.6 K, the parallel connection exhibited a SCP of 2.5 W g^−1^ (with Δ*T*_c_ of 2.4 K) and a system COP of 3.7 (with Δ*T*_c_ of 0.9 K). Compared with the elastocaloric cooling prototypes (associated with heat transfer fluid) driven by tension or compression (Fig. 5a), our prototype using the coil-bending actuation has the advantages of a high *A*_spht_ of 12.6 cm^2^ g^−1^ and a very low *F*_sp_ of 26 N g^−1^, which substantially reduces the size of motors and supporting structures, and therefore enables a miniature design. The standalone air-cooling prototype, including all components (actuators, transmission components, a pump, elastocaloric materials, and a controller), had dimensions of 300×250×200 mm^3^ (refer to Supplementary Fig. S6). Thanks to the design of the coil-bending mode and the parallel/serial air channels, the Δ*T*_c_ of 10 K and the SCP of 2.5 W g^−1^ of our air cooler exceeded the performance of reported caloric air-cooling prototypes and demos^23,31,38,39,49,57–59^ (Fig. 5b).

## Discussion

We further discuss possible improvements to the cooling performance of our coil-bending elastocaloric air cooler. Active regeneration^28,40^ has not been implemented in our coil-bending elastocaloric air cooler at present. The maximum temperature drop of the outlet airflow of our cooler is equivalent to the adiabatic temperature change of the NiTi wires. Nevertheless, it is feasible to achieve a similar effect as the active regeneration by incorporating a multi-stage cooling configuration. This configuration leverages the cooling capacity from the previous stage to pre-cool the NiTi wires and lead screws of the subsequent stage, thereby increasing the overall temperature drop of the outlet airflow. To substantially increase the SCP of the air cooler, we propose increasing the specific heat transfer area and the operating frequency. One effective way to increase the specific heat transfer area and enhance the heat transfer efficiency is to reduce the diameter of NiTi wires. Meanwhile, a smaller diameter directly reduces the number of defects in the material, thereby reducing crack nucleation and increasing fatigue life. Additionally, utilizing advanced fatigue-resistant SMA can mitigate fatigue-related concerns. An example of such promising SMA for elastocaloric cooling is Ti_52.8_Ni_22.2_Cu_22.5_Co_2.5_, which exhibits an ultra-high fatigue life of over 20 million cycles under the maximum tensile stress of 480 MPa^60^. The total cooling power of the elastocaloric air cooler is directly influenced by the amount of NiTi used. In the current configuration, only 6 pieces of NiTi wires are utilized. Nevertheless, by incorporating a longer lead screw and expanding the number of tube sections, we can accommodate more NiTi wires. Additionally, each motor in the current setup only drives one lead screw. To increase the driving efficiency and reduce costs, we can incorporate a one-to-multi gear box, allowing a single-shaft motor to simultaneously drive multiple lead screws that rotate in the same direction. This modification will effectively increase the amount of NiTi wires being driven, thereby resulting in a higher cooling power output. Personal space cooling devices, such as air conditioners and refrigerators, typically employ air as the terminal outlet medium. However, most existing caloric cooling prototypes use water as the heat transfer fluid and neglect the nontrivial heat transfer loss between water and air. The major advantage of our air cooler is that it uses NiTi wires/ribbons to directly cool the air and achieves a substantial temperature drop of 10.6 K and a system COP of 3.7. We believe that there is substantial potential to enhance the energy efficiency in elastocaloric prototypes by implementing the recovery of the unloading (uncoiling) work, as demonstrated in previous studies utilizing uniaxial loading techniques^28,32,53^. There are two potential methods for storing and recovering the rotational mechanical work. The first method involves directly storing the rotational work in clockwork springs or flywheels, allowing the stored energy to be utilized for loading samples in subsequent cycles. The second method involves converting the rotational mechanical work into electrical energy using electric generators and storing it in batteries. This approach, known as regenerative braking, is widely employed in battery electric vehicles. Both methods exhibit substantial potential for improving the energy efficiency of coil-bending-based elastocaloric air coolers.

In summary, we developed an easy-to-drive and energy-efficient elastocaloric air cooler based on coil-bending actuation. The low specific driving force of NiTi wires under the coil-bending was only 26 N g^−1^, less than a fifth of that of uniaxial tension/compression. By pumping air through the constant cold zone comprised of uncoiled NiTi wires, our elastocaloric air cooler achieved a continuous cold outlet airflow with an average temperature drop of 10.6 K (the amplitude of temperature fluctuation is 0.95 K), equivalent to 83% of the adiabatic temperature drop of NiTi wires (12.8 K). Our cooler’s specific cooling power and system coefficient of performance reached 2.5 W g^−1^ and 3.7, respectively, thanks to the large specific heat transfer area of 12.6 cm^2^ g^−1^ of NiTi wires. More importantly, our air-cooling prototype is a standalone system with all components integrated into compact dimensions of 300 × 250 × 200 mm^3^. The results demonstrate the potential of coil-bending actuation in bringing us one step closer to commercializing elastocaloric cooling technology.

## Methods

### Materials and sample preparation

The NiTi ribbons with gauge sizes of 1.5 × 300 mm^2^ (see Supplementary Note S1) were wire-cut from a 0.50-mm-thick NiTi sheet (Johnson Matthey Corporation) with a chemical composition of Ni_56_Ti_44_ in mass fraction and an austenitic finish temperature of 274 K. A dog-bone-shaped specimen with gauge sizes of 2 × 10 mm^2^ for tensile tests was cut from the NiTi sheet. The *ϕ*0.50 mm and 325 mm-long round NiTi wires (Kellogg’s Research Labs Corporation) used in the elastocaloric air cooler had an austenitic finish temperature of 278 K and the same chemical composition and microstructure as the ribbons.

To obtain a stable cyclic phase transformation behavior, the NiTi ribbons and wires were trained for 200 coil-bending cycles at room temperature before the measurement of elastocaloric cooling performance. The loading/unloading time was set as 60 s to ensure an isothermal condition. The maximum tensile strain at the surface of the coil-bent ribbons/wires was 7%. The evolution of adiabatic temperature changes of the NiTi wires with the training cycle is shown in Supplementary Note S5. Similarly, the NiTi dog-bone specimen was trained^61–63^ for 200 tensile cycles with a strain range of 7% at a strain rate of 7 × 10^−4^ s^−1^.

### Characterisation of elastocaloric performance

The structures and dimensions of the lead screws are detailed in Supplementary Note S1 and S2. The characterisation of elastocaloric performance was carried out at room temperature of 299 K. Three lead screws with radius of root circle (*r* = 4.5 mm, 7 mm, and 9 mm) and corresponding maximum surface tensile strain (*ε*_max_ = 7.01%, 4.28%, and 2.88%) of the coil-bent NiTi ribbon were tested. The NiTi mechanical properties under bending deformation can be referred to in previous literatures^64–66^. The experimental setups are shown in Supplementary Note S3. The NiTi ribbon was initially coiled on one lead screw. During the operation, the ribbon was uncoiled from the lead screw and coiled onto another one in 0.5 s. It was then held in position for 100 s. The surface temperature of the NiTi ribbon in operation was captured by an infrared camera (FLIR SC7700M). The output torque *τ* of the motors was measured by a torque sensor. The driving force *F* was calculated as *τ*/*r*. The specific driving force *F*_sp_ was calculated by dividing the *F* with the mass of the NiTi ribbon (see Supplementary Note S8). For comparison, the NiTi dog-bone was loaded in 0.5 s to the required strain (7.01%, 4.28%, and 2.88%) and held at the strain for 100 s by a universal testing machine (MTS), where the temperature and applied load on the dog-bone were recorded. The dog-bone was then unloaded in 0.5 s to measure the temperature drop and held for 100 s for heat transfer. The *F*_sp_ was calculated as the applied load divided by the mass of the dog-bone (*F/m*).

### Operating conditions and performance metrics calculations of the air cooler

The air channel had an inner diameter of 2 mm. Airflow was pumped into the silicon tubes and cooled by the uncoiled NiTi wires. The cooling performance was evaluated at the operating frequencies *f* of 0.048 Hz, 0.071 Hz, 0.143 Hz, and 0.286 Hz. The elastocaloric air cooling cycle was composed of four stages: forward rotation, holding, reverse rotation, and holding. The duration of each stage at different *f* is shown in Supplementary Tab. S3.

Since it is difficult to build an adiabatic environment for the cold airflow and apply a thermal load to measure the cooling power of our air cooler, we calculated the cooling power based on the average temperature drop (Δ*T*_c_) of the airflow in the steady state. The temperatures of the inlet and the outlet airflow were recorded in-situ by K-type thermocouples. The specific cooling power (SCP) of the air cooler was calculated by Eq. (1):

1 $$SCP=\frac{{{c}}_{{{{{{\rm{air}}}}}}}\cdot {{\rho }}_{{{{{{\rm{air}}}}}}}\cdot {\omega }\cdot \varDelta {{{{{{\rm{T}}}}}}}_{{{{{{\rm{c}}}}}}}}{{{m}}_{{{{{{\rm{NiTi}}}}}}}}$$

where *ρ*_air_ = 1.205 kg m^−3^ is the air density; *c*_air_ = 1005 J kg^−1^ K^−1^ is the specific heat capacity of air; *ω* is the flow rate of the outlet airflow; *m*_NiTi_ is the mass of the NiTi wires in the air channels.

The coefficient of performance (COP) of the air cooler was defined as the ratio of the SCP to the specific input mechanical power ${\dot{{w}}}_{{{{{{\rm{mech}}}}}}}$, as shown in Eq. (2). Since the two lead screws rotated in the same direction at the same speed, only one of the two motors was required to drive the coiling and uncoiling of NiTi wires during forward and reverse rotation. The average specific torque change $\overline{{\Delta {{{{{\rm{\tau }}}}}}}_{{{{{{\rm{sp}}}}}}}}$ was used to calculate the ${\dot{{w}}}_{{{{{{\rm{mech}}}}}}}$ in Eq. (3). The detailed analysis of ${\dot{{w}}}_{{{{{{\rm{mech}}}}}}}$ is shown in Supplementary Note S9. It should be noted that the power of the air pump was not considered when calculating the COP. However, it is expected that the inclusion of the pump power would result in a reduction of 10%-20% in the system COP^4^.

2 $$COP=\frac{SCP}{{\dot{w}}_{{{{{{\rm{mech}}}}}}}}$$

3 $${\dot{w}}_{{{{{{\rm{mech}}}}}}}=\overline{\varDelta {\tau }_{{{{{{\rm{sp}}}}}}}}\cdot 2\pi \cdot {n}_{{{{{{\rm{rps}}}}}}}\cdot \frac{{P}_{{{{{{\rm{r}}}}}}}}{P}$$

where *n*_*rps*_ is the number of rotations per second; the angular velocity is 2π*n*_*rps*_; *P*_r_ is the sum of duration of the forward rotation and the reverse rotation in each operating cycle; *P* is the duration of one operating cycle.

### Supplementary information

Supplementary Information

Supplementary Movie

Description of Additional Supplementary Files

### Source data

Source Data

## Data Availability

The data that support the findings of this study have been included in the main text and Supplementary Information. All other relevant data supporting the findings of this study are available from the corresponding authors upon request. Source data are provided with this paper.
