# Supplementary material for: Continuous and efficient elastocaloric air cooling by coil-bending
Source: Nat Commun. 2023 Dec 2;14:7982. doi: 10.1038/s41467-023-43611-6 (PMC10693641; doi:10.1038/s41467-023-43611-6)
Supplement: Supplementary file 1 — Supplementary Information [file 41467_2023_43611_MOESM1_ESM.pdf]

Continuous and efficient elastocaloric air cooling by coil-bending

Xueshi Li[a], Peng Hua[a, b, *], Qingping Sun[a, b, *]

[a] *Department of Mechanical and Aerospace Engineering, The Hong Kong University of Science and Technology, Kowloon, Hong Kong, China*

[b] *HKUST Shenzhen-Hong Kong Collaborative Innovation Research Institute, Futian, Shenzhen, Guangdong, China*

*Corresponding authors

Email addresses: penghua@ust.hk (P. Hua), meqpsun@ust.hk (Q. Sun)

Note S1: Structure design of the lead screw

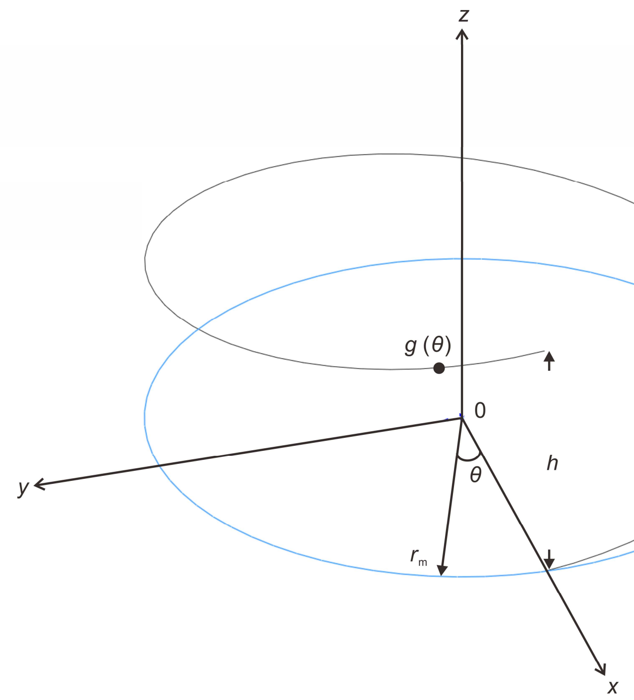

Fig. S1. Schematic helix of lead screw in a Cartesian coordinate system.

The lead screw has a helix structure, which is described by a parametric equation g(θ) shown in Eqs. (s1) and (s2),

$$g(\theta)=\left[x(\theta),y(\theta),z(\theta)\right] \tag{s1}$$

$$\begin{cases} x(\theta)=r_{\mathrm{m}}\cos\theta \\ y(\theta)=r_{\mathrm{m}}\sin\theta \\ z(\theta)=\alpha\theta \end{cases} \tag{s2}$$

where r_{m} is the radius of based circle in the helix, θ is the angle. Coefficient α in the $z(\theta)$ is calculated by the following equations,

$$\begin{cases} z(\theta)=\alpha\theta \\ z(0)=0 \\ z(2\pi)=h \end{cases} \tag{s3}$$

$$\alpha=\frac{z(2\pi)-z(0)}{2\pi-0}=\frac{h}{2\pi} \tag{s4}$$

where h is helical pitch.

The curvature κ of $g(\theta)$ is determined by using the Eq. (s5),

$$\kappa = \frac{|g' \times g''|}{|g'|^3} = \frac{\sqrt{(z''y' - y'z')^2 + (x''z' - z''x')^2 + (y''x' - x''y')^2}}{(x'^2 + y'^2 + z'^2)^{3/2}} \tag{s5}$$

where required expressions are shown as follow.

$$\begin{cases} x'(\theta) = -r_{\mathrm{m}} \sin\theta \\ x''(\theta) = -r_{\mathrm{m}} \cos\theta \\ y'(\theta) = r_{\mathrm{m}} \cos\theta \\ y''(\theta) = -r_{\mathrm{m}} \sin\theta \\ z'(\theta) = \dfrac{h}{2\pi} \\ z''(\theta) = 0 \end{cases} \tag{s6}$$

Thus, κ can be derived as the following.

$$\begin{aligned} \kappa &= \frac{\sqrt{(z''y' - y'z')^2 + (x''z' - z''x')^2 + (y''x' - x''y')^2}}{(x'^2 + y'^2 + z'^2)^{3/2}} \\ &= \frac{\sqrt{\left[-(-r_{\mathrm{m}} \sin\theta)\cdot\frac{h}{2\pi}\right]^2 + \left[(-r_{\mathrm{m}} \cos\theta)\cdot\frac{h}{2\pi}\right]^2 + \left[(-r_{\mathrm{m}} \sin\theta)^2 + (r_{\mathrm{m}} \cos\theta)^2\right]^2}}{\left[(-r_{\mathrm{m}} \sin\theta)^2 + (r_{\mathrm{m}} \cos\theta)^2 + \left(\frac{h}{2\pi}\right)^2\right]^{3/2}} \\ &= \frac{\sqrt{\left(\frac{h}{2\pi}\right)^2 r_{\mathrm{m}}^{\ 2} + r_{\mathrm{m}}^{\ 4}}}{\left[\left(\frac{h}{2\pi}\right)^2 + r_{\mathrm{m}}^{\ 2}\right]^{3/2}} \\ &= \frac{r_{\mathrm{m}}\sqrt{\left(\frac{h}{2\pi}\right)^2 + r_{\mathrm{m}}^{\ 2}}}{\left[\left(\frac{h}{2\pi}\right)^2 + r_{\mathrm{m}}^{\ 2}\right]^{3/2}} \\ &= \frac{r_{\mathrm{m}}}{\left(\frac{h}{2\pi}\right)^2 + r_{\mathrm{m}}^{\ 2}} \end{aligned} \tag{s7}$$

The radius of curvature r_{c} at one point in the helix can be calculated by the Eq. (s8),

$$r_{\mathrm{c}} = \frac{1}{\kappa} = \frac{\left(\frac{h}{2\pi}\right)^2 + r_{\mathrm{m}}^{\ 2}}{r_{\mathrm{m}}} \tag{s8}$$

where the r_m is calculated from the radius of root circle on the lead screw (r). When NiTi ribbon is coiled onto the lead screw, the r_m is equal to $r + 1/2\ t_{NiTi}$, where t_{NiTi} is ribbon thickness (as shown in Fig. S2a). After selecting h and t_{NiTi}, the r_c in the geometric centre of bent NiTi ribbon can be calculated by Eq. (s8), where it reflects the bending deformation degree for the thin ribbon[1]. Here, lead screws with the same h of 4 mm and three different radiuses of root circle (r = 4.5 mm, 7 mm, 9mm) were designed and fabricated. The two ends of the NiTi ribbon were fixed on the two lead screws (Fig. S2b). The groove on the lead screw avoided the overlapping of NiTi ribbons during operation. The groove height was designed to be larger than the t_{NiTi}. It should be noted that the calculation of r_c assumes pure bending of NiTi ribbons.

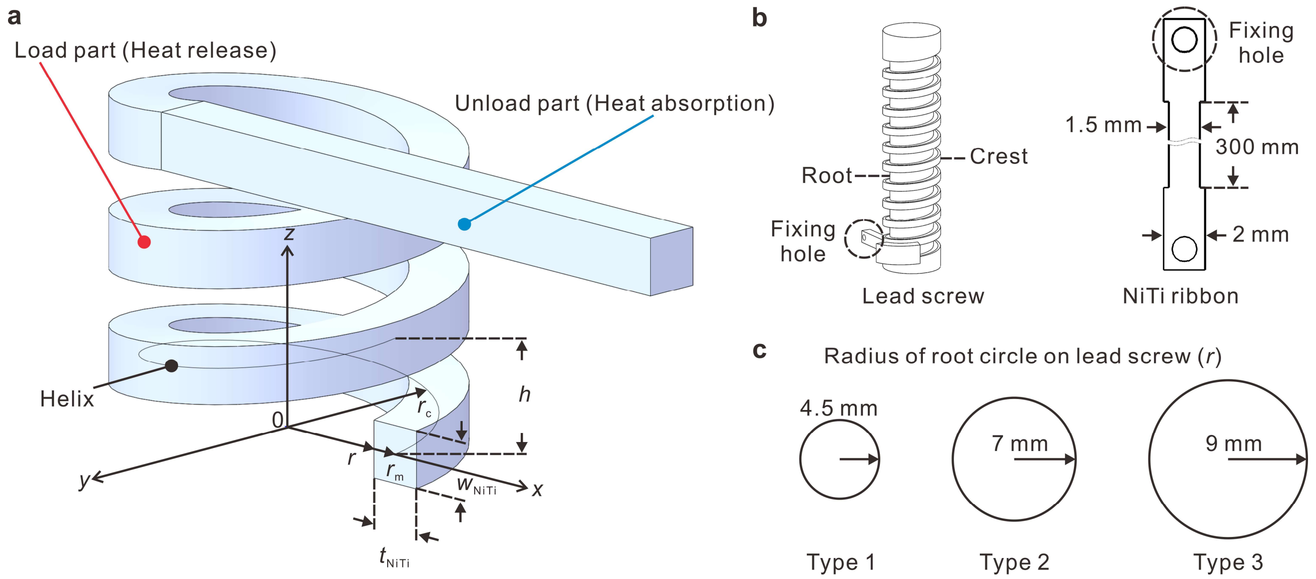

Fig. S2. **a** A NiTi ribbon under the coil-bending. **b** The structure of a lead screw and the dimensions of NiTi ribbon. **c** Three lead screws with different radiuses of root circle.

Note S2: Maximum surface tensile strain of NiTi under coil-bending

The neutral axis shifts during the bending of NiTi ribbons due to the tension/compression asymmetry[2,3]. It usually shifts towards the compressive side to reach an equilibrium of the moments arising from the axial tensile and compressive stresses. Theoretical values of the maximum surface tensile strain in pure bending ($\varepsilon_{max,\,pure}$) are calculated by Eq. (s9) and (s10),

$$\varepsilon_{max,\,pure} = \frac{r_t}{r_c} \tag{s9}$$

$$r_c = r_t + r^* \tag{s10}$$

where r_t is half the ribbon thickness, r^* is the radius of based cylinder fabricated by the resin 3D printing, r_c is the radius of curvature.

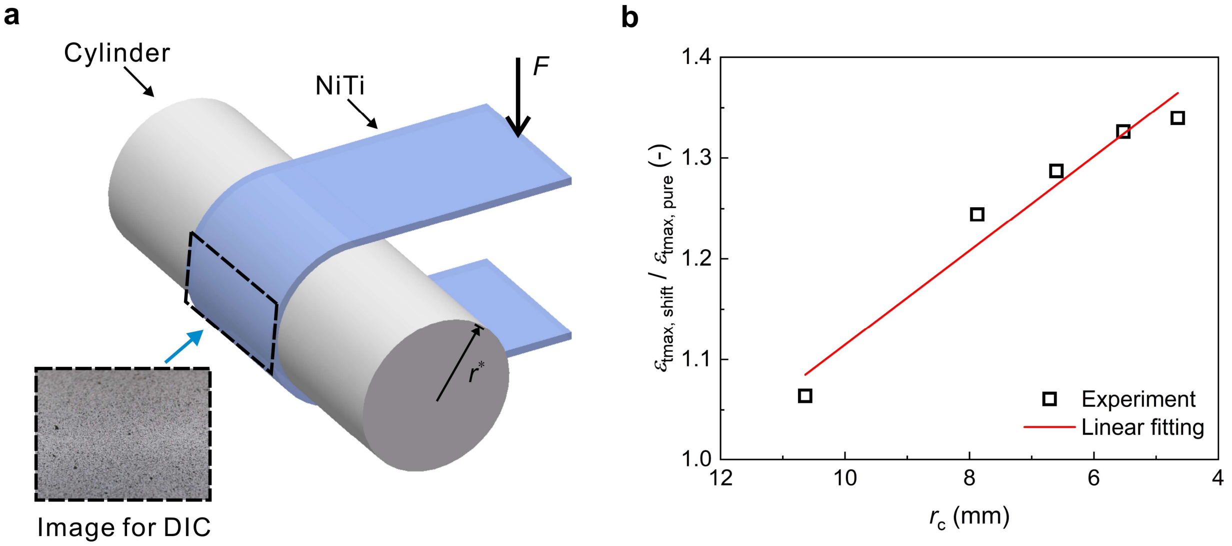

Fig. S3. a DIC setup for measurement of maximum surface tensile strain of NiTi under pure bending. **b** The shifting ratio of maximum surface tensile strain versus r_c.

The actual maximum surface tensile strain of NiTi $\varepsilon_{max,\,shift}$ is measured by digital image correlation (DIC), as shown in Fig. S3a. The ratios of the $\varepsilon_{max,\,shift}$ and $\varepsilon_{max,\,pure}$ under different r_c are shown in Fig. S3b. The ratio follows a linear relation with r_c. Thus, the $\varepsilon_{max,\,shift}$ of NiTi ribbon under coil-bending

can be estimated by the linear fitting equation. To minimize the functional degradation of NiTi, the specimens were trained before the testing.

Note S3: Experimental setup

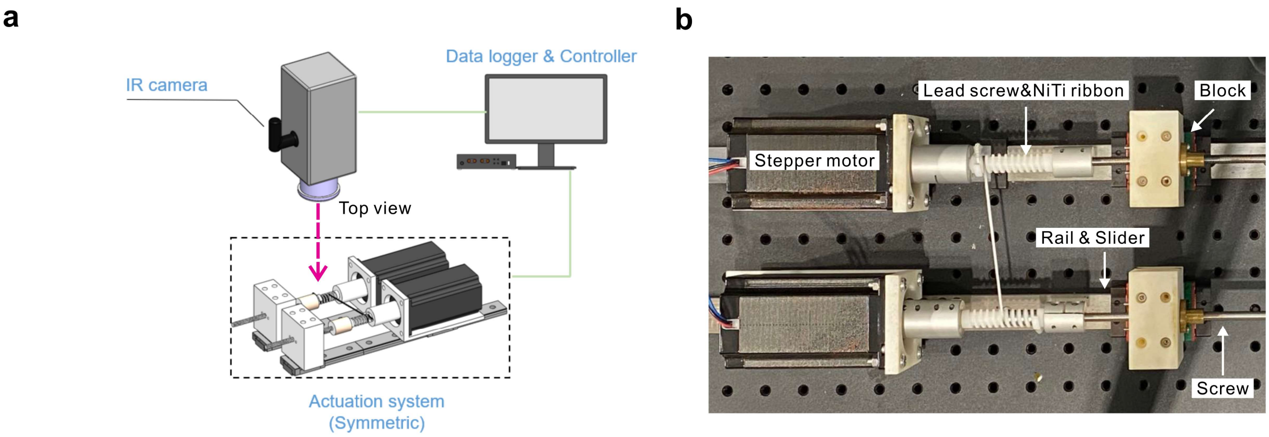

Fig. S4. a Experimental setup for measuring the adiabatic temperature change of NiTi ribbon. **b** Photo of the actuation system.

Experimental setup for measuring the adiabatic temperature change of a NiTi ribbon under coil-bending is shown in Fig. S4a. An infrared camera (FLIR SC7700M) was used to record the surface temperature evolution of the NiTi ribbon under coil-bending. The actuation system was based on two stepper motors (Samsr, SS2305A40A) controlled by Arduino. The maximum static torque of the stepper motor was 3.0 N·m. As shown in Fig. S4b, the actuation system had a symmetric structure of two units, where each unit was composed of a stepper motor, a lead screw, an end screw, and a block aligned on a rail in series. A helical pitch of 4 mm was used for the internal thread of the block, the end screw, and the lead screw. The stepper motors were installed on sliders to enable linear motion along the rail during the coil-bending of NiTi ribbon. The sliders, lead screws (Fig. S5) and blocks were fabricated by 3D printing of photosensitive resin. Initially, half length of the NiTi ribbon was coiled onto the two symmetric lead screws as shown in Fig. S4b. The uncoiled NiTi ribbon was located between the two lead screws and formed a constant cold zone that remained at the same position during operation.

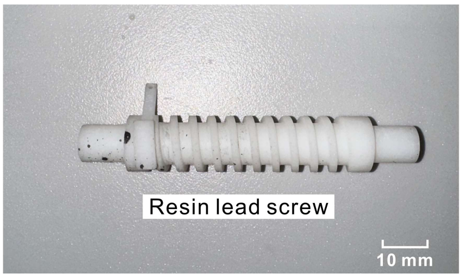

Fig. S5. The resin lead screw.

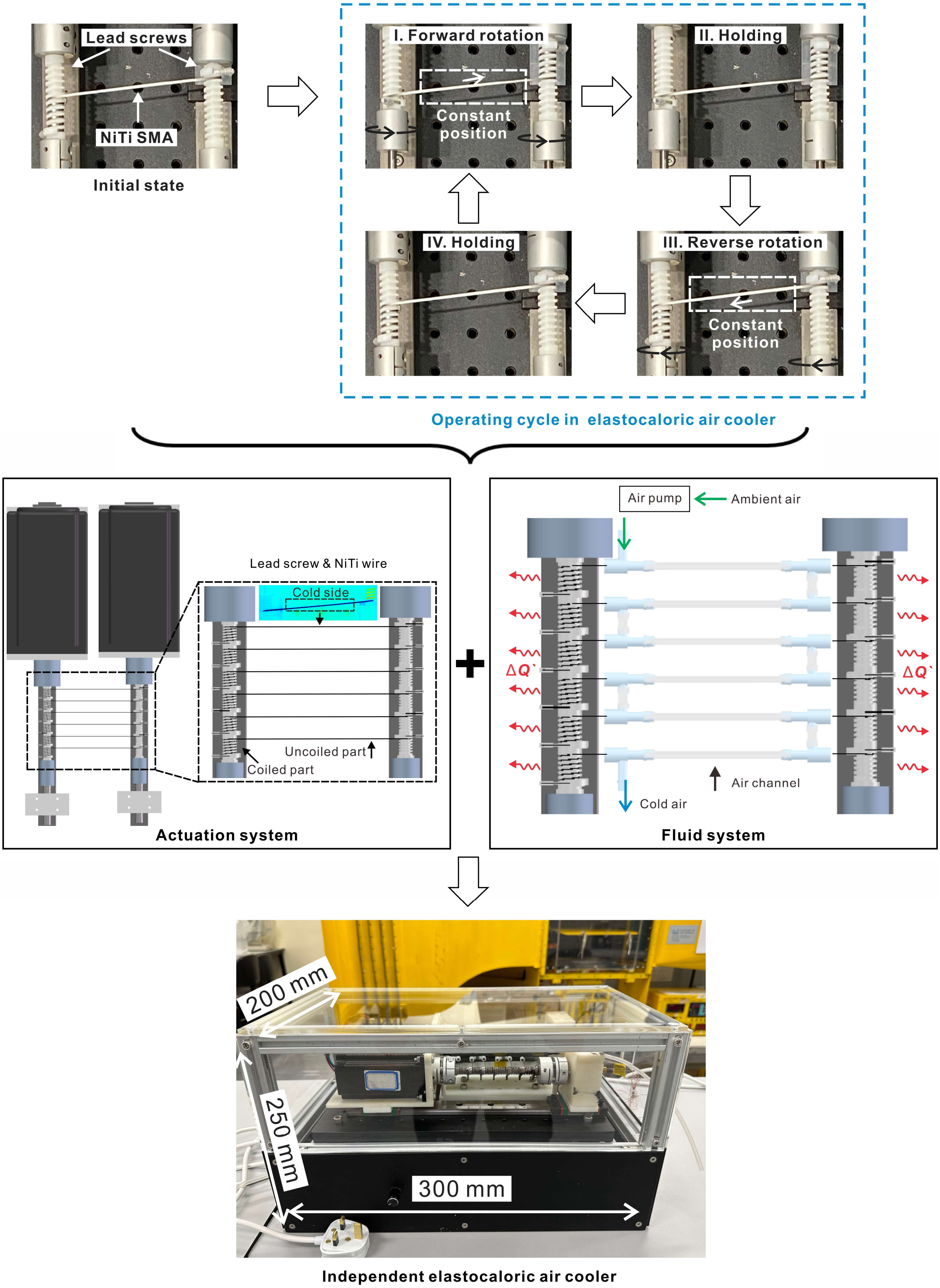

Fig. S6. An overview of the elastocaloric air cooler based on the coil-bending of NiTi wires.

Fig. S6 shows an overview of the elastocaloric air cooler based on the coil-bending of NiTi wires. Steel lead screws (Fig. S7a) with *r* of 4.5 mm and a helical pitch of 2 mm were used to increase the heat conduction of the hot zone. Six NiTi wires (325 mm long; 0.5 mm in diameter) were used in the serial connection, where both ends of the NiTi wires were fixed on the lead screws. The uncoiled part of the NiTi wires was sealed in air channels (Fig.S7b), where each section had an effective heat transfer length of 80 mm. Room-temperature air was pumped through the air channel by an independent mini-pump, which provided a stable airflow with a flow rate of up to 10 L·min^{-1}. A flowmeter (MF4708-B3-10-BV-A) was used to measure the flow rate of the outlet air ω. Type-K thermocouples (OMEGA co.) with a diameter of 0.08 mm were placed at the inlet and outlet of the connected air channels to measure the temperature of airflow. In the constant cold zone, heat was transferred from the air to the cold uncoiled NiTi wires by convection. In the hot zone, heat was mostly transferred from the hot coiled NiTi wires to the steel lead screws by conduction (the characteristic heat transfer time of the NiTi wire is shown in Note S5).

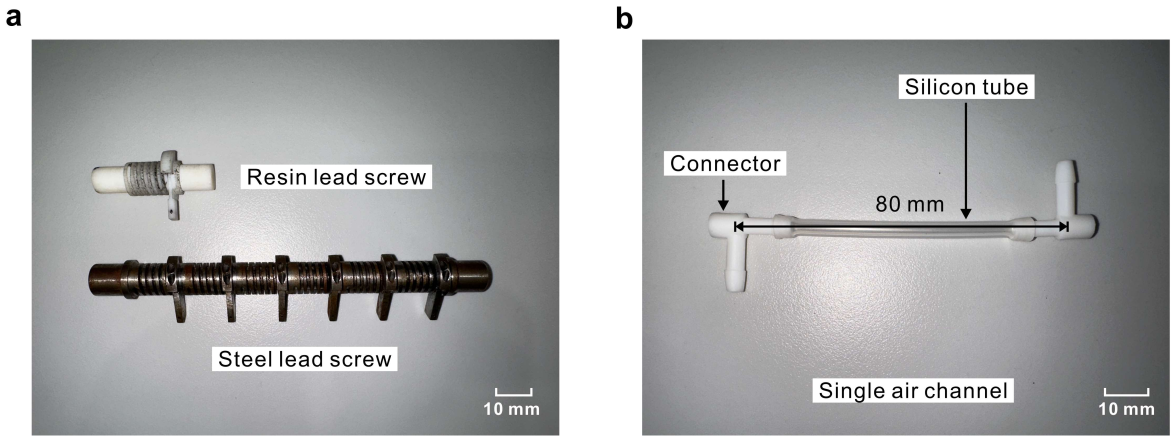

Fig. S7. a The steel lead screw and resin lead screw used in the elastocaloric air cooler. **b** A single air channel used in the elastocaloric air cooler.

The temperature difference between the inlet air and the outlet air was calculated as shown in Fig. S8. It should be mentioned that all tests were performed at room temperature (298 K). The temperature drop of air increases with the decrease in the air flowrate, as a lower flowrate allows more heat transfer time. The average temperature drop of air in the steady state ΔT_c is used to calculate the specific cooling power of the air cooler (*SCP*).

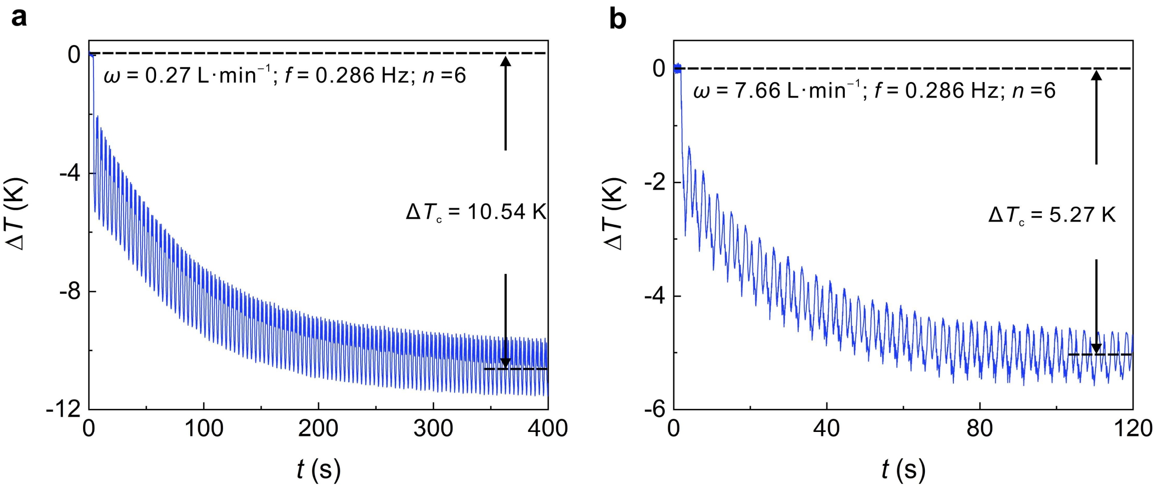

Fig. S8. Temperature drop of the air versus the operating time, **a** At the flow rate of 0.27 $L \cdot min^{-1}$; **b** At the flow rate of 7.66 $L \cdot min^{-1}$, where *f* is operating frequency and *n* is the number of installed air channels.

As shown in Fig. S8, the actual temperature change of the outlet airflow has temperature fluctuations, due to the motion of the NiTi wires and the variations in the flow rate. The temperature fluctuation of the outlet airflow is determined as the ΔT_{fluct}, which is twice the temperature fluctuating amplitude in the steady state. The ΔT_{fluct} decreases with the airflow rate and the operating frequency (*f*), as shown in Fig. S9.

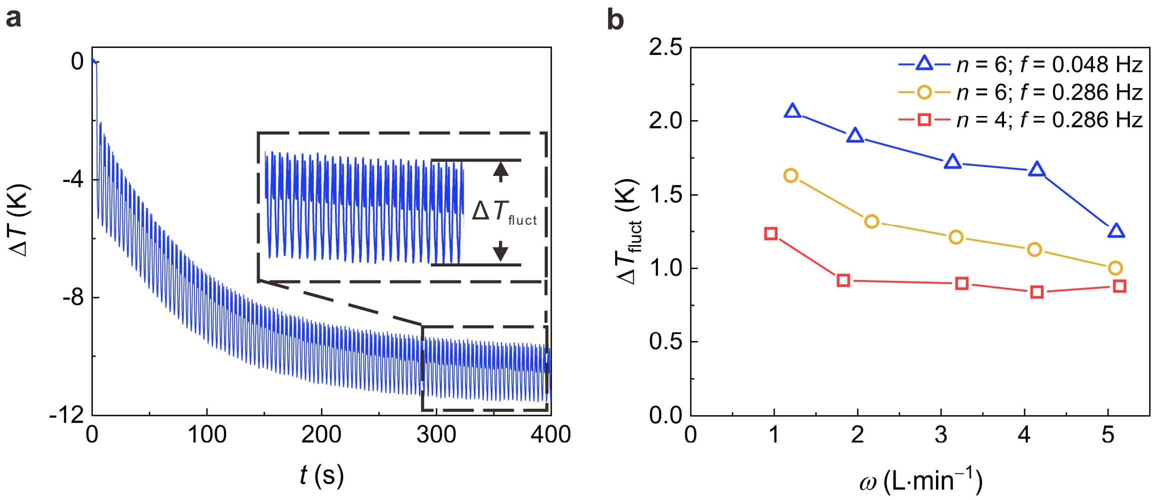

Fig. S9. **a** Determination of the temperature fluctuation of the outlet airflow ($\Delta T_{\mathrm{fluct}}$) of the elastocaloric air cooler. **b** $\Delta T_{\mathrm{fluct}}$ versus the flow rate (ω).

Note S4: Analysis of uncertainty in measurements

Standard experimental uncertainty analysis was performed using two evaluation methods[4]. A summary of the experimental uncertainty analysis is given in Tab.S1. Type A (u_A) standard uncertainty is obtained from a probability density function derived from an observed frequency distribution. Type B (u_B) standard uncertainty is obtained from an assumed probability density function based on the degree of belief that an event will occur. The standard uncertainty of a directly measured result is termed combined standard uncertainty and denoted by $u_{C,d}$. The standard uncertainty of an indirect experimental result ($u_{C,i}$) is evaluated by Eq. (s14),

$$u_A(x) = \sqrt{\frac{1}{n(n-1)}\sum_{i=1}^{n}(x_i - \bar{x})^2} \tag{s11}$$

$$u_B(x) = \frac{\sqrt{3}}{3}\Delta_i \quad \text{or} \quad u_B(x) = \frac{\sqrt{3}}{6}\delta_x \tag{s12}$$

$$u_{C,d}(x) = \sqrt{[u_A(x)]^2 + [u_B(x)]^2} \tag{s13}$$

$$\frac{u_{C,i}(q)}{|q|} = \sqrt{\left[\frac{u_{C,d}(x)}{x}\right]^2 + \left[\frac{u_{C,d}(y)}{y}\right]^2 + \cdots \left[\frac{u_{C,d}(z)}{z}\right]^2}, q = f(x, y, \cdots z) \tag{s14}$$

where x, y, and z are the directly measured experimental results; q is the indirect experimental result calculated from the direct results; n is the number of measurements; Δ_i is the maximum permissible error of the instrument; δ_x is the resolution of the instrument. The uncertainty analyses of experiment results are given in the following five sections.

1. Airflow rate (ω), Temperature of airflow (T_{air}), mass of NiTi (m_{NiTi}), and specific cooling power (SCP):

Airflow rate (ω) was measured by an air flowmeter, and 3 rounds of independent measurements were performed. The u_A of ω is 0.012 L·min^{-1}, while the u_B of u is 0.023 L·min^{-1} (Δ_i of the air

flowmeter is 0.04 L·min^{-1}). Thus, the $u_{C,d}$ of u is 0.026 L·min^{-1}. Inlet and outlet temperature of airflow (T_{air}) were measured by the Type-K thermocouple in 3 rounds of independent measurements. The temperature difference between the inlet and outlet airflow was used to calculate the *SCP*. The u_A of T_{air} is 0.03 K. The u_B of T_{air} is 0.003 K (δ_x of the NI acquisition card is 0.01 K), and the $u_{C,d}$ of T_{air} is 0.03 K. Mass of NiTi wire (m_{NiTi}) was measured by the electronic weight scale. The u_A of m_{NiTi} is 1.77×10^{-4} g calculated from the results of 3 rounds of independent measurements, the u_B of m_{NiTi} is 3×10^{-4} g (δ_x of the electronic weight scale is 0.001 g), and the $u_{C,d}$ of m_{NiTi} is 3.48×10^{-4}g. The specific cooling power (*SCP*) of cooler is related by the ω, T_{air}, and m_{NiTi} (here, the density ρ and specific heat capacity c of air are considered as constant values in the condition of experiments). Thus, the $u_{C,i}$ (0.002 W·g^{-1}) of *SCP* can be calculated by Eq. (s14).

2. Surface temperature of NiTi (T_{NiTi}) measured by the infrared camera:

Surface temperature of NiTi wires (T_{NiTi}) was captured by an infrared camera. Four rounds of measured results were used to calculate the u_A of T_{NiTi} by Eq. (s11), which gives a value of 0.055 K. The u_B of T_{NiTi} is 0.003 K (δ_x of the infrared camera is 0.01 K). The $u_{C,d}$ of T_{NiTi} is 0.082 K by Eq. (s13).

3. Actuating force (F) and strain (ε) of NiTi dog-bone specimen were measured by a universal testing machine:

Actuating force (F) and strain (ε) of a NiTi dog-bone specimen were measured by a universal testing machine. The tensile strain of the NiTi dog-bone specimen was measured by an extensometer. The results of 3 rounds of independent measurements were used to calculate u_A. The u_A of F is 1.84 N and the u_A of ε is 5.18×10^{-3}%. According to the Δ_i of the testing machine (1.00 N and 0.01%), the u_B of F

is 0.87 N and the u_B of ε is 0.009%. The combined standard uncertainties can be calculated by Eq. (s13), the $u_{C,d}$ of F is 2.03 N and the $u_{C,d}$ of ε is 0.01%.

4. Surface strain of NiTi sheet (ε_{DIC}) by digital image correlation (DIC):

The deformation of a NiTi sheet was captured by a camera, where the strain field of the NiTi sheet (ε_{DIC}) was calculated by a software package. The strain results of 4 rounds of independent measurements were recorded. The u_A of ε is calculated as 0.024% by Eq. (s11). The u_B of ε is 0.003% (δ_x of the DIC software is 0.01%). The $u_{C,d}$ of ε is 0.024% by Eq. (s13).

5. Torque (τ) measured by the torque sensor, specific mechanical input power ($\dot{w}_{mech}$), and coefficient of performance (COP):

The torque (τ) was measured by the torque sensor, as detailed in Note S6. According to the results of 3 independent measurements of the elastocaloric air cooler in operation, the u_A of τ is 7.33×10^{-4} N·m and the u_B of τ is 0.003 N·m (δ_x of the torque sensor is 0.01 N·m). The $u_{C,d}$ of τ is 0.003 N·m by Eq. (s13). The specific mechanical input power ($\dot{w}_{mech}$) of cooler scales with the τ. The $u_{C,i}$ of $\dot{w}_{mech}$ is calculated as 0.027 $W\cdot g^{-1}$ by Eq. (s14). The $u_{C,i}$ of coefficient of performance (COP) is 0.024 by Eq. (s14).

Tab. S1. Summary of the experimental uncertainty analysis.

	u_A	u_B	$u_{C,d}$	$u_{C,i}$	uncertainty
ω	0.012 L·min^{-1}	0.023 L·min^{-1}	0.026 L·min^{-1}	/	0.64%
T_{air}	0.03 K	0.003 K	0.03 K	/	1.54%
m_{NiTi}	1.77×10^{-4} g	3×10^{-4} g	3.48×10^{-4} g	/	0.09%
SCP	/	/	/	0.002 W·g^{-1}	1.96%
T_{NiTi}	0.055 K	0.003 K	0.055 K	/	0.02%
F	1.87 N	0.87 N	2.03 N	/	0.34%
ε	5.18×10^{-3}%	0.009%	0.01%	/	0.33%
ε_{DIC}	0.024%	0.003%	0.024%	/	0.52%
τ	7.33×10^{-4} N·m	0.003 N·m	0.003 N·m	/	7.44%
$\dot{w}_{mech}$	/	/	/	0.027 W·g^{-1}	7.59%
COP	/	/	/	0.024	7.81%

Note S5: Characterisation of the elastocaloric effect of NiTi wires

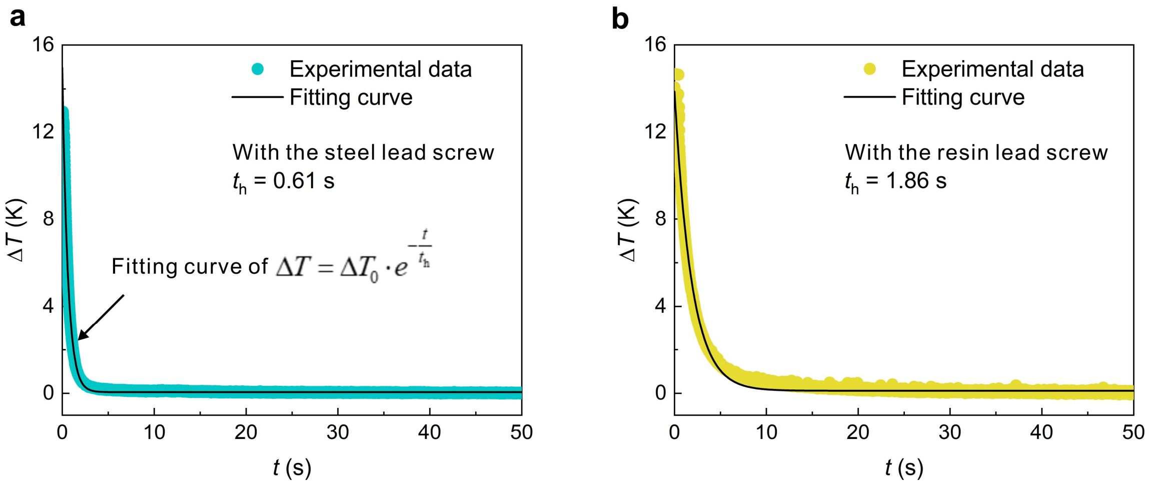

Fig. S10. Temperature relaxations of the hot NiTi wire in still air in contact with **a** the steel lead screw and **b** the resin lead screw.

To characterize the elastocaloric effect, we measured the adiabatic temperature changes of NiTi wires used in the elastocaloric air cooler. Two lead screws made of different materials were used. A steel lead screw was used in the air cooler to reduce the heat accumulation, while a resin lead screw was used to measure the adiabatic temperature changes of NiTi wires. Both lead screws had a radius of root circle r of 4.5 mm. The temperature relaxations of a NiTi wire in the hot zone are shown in Fig. S10. It should be noted that the NiTi wires used in this test were trained 200 cycles before testing. The motors quickly rotated 3 turns in 0.5 s to ensure the adiabatic condition, and the surface temperature of the NiTi wire was recorded using an infrared camera. The temperature jumps were 12.9 K and 14.6 K, for the steel lead screw and resin lead screw, respectively. The steel lead screw had a higher thermal conductivity, resulting in a notable heat conduction loss and low temperature jump. The characteristic heat transfer time (t_h) was obtained by fitting the experimental data with the relaxation equation Eq. (s15) in still air[5].

$$\Delta T = \Delta T_0 \cdot e^{-\frac{t}{t_h}} \tag{s15}$$

The characteristic heat transfer time (t_h) of the NiTi wire in contact with the steel lead screw was 0.61 s, which is much lower than the smallest half cycle (1.75 s at the frequency of 0.286 Hz). We used the steel lead screw to increase the thermal conduction rate and reduce the effect of the hot side on the cooling performance of our air cooler. Thus, the released heat of the coiled NiTi wire can be sufficiently transferred to the steel lead screw and eventually to the surrounding environment, resulting in the high cooling power of the air cooler. In contrast, the t_h of the NiTi wire in contact with the resin lead screw reached 1.86 s, due to the low thermal conductivity of resin. Therefore, we used the resin lead screw to measure the adiabatic temperature jump of the NiTi wire under coil-bending.

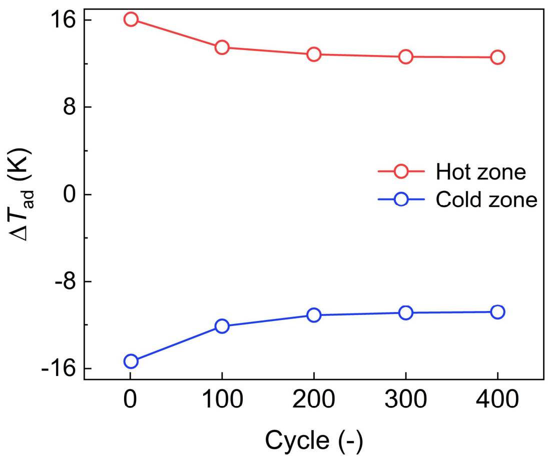

Fig. S11. Adiabatic temperature changes of the NiTi wire during training.

The received NiTi wire initially showed an unstable functional behaviour. Thus, the NiTi wire was trained in quasi-isothermal coiling-uncoiling cycles to stabilize the functional behaviour. The number of rotations per second was set as 1/12 s^{-1} to ensure the quasi-isothermal condition. The adiabatic temperature changes of the NiTi wire were recorded during the training process, as shown in Fig. S11. The functional behaviour of the NiTi wire was stable after the training for 200 cycles.

Note S6: Heat transfer processes in the elastocaloric air cooler

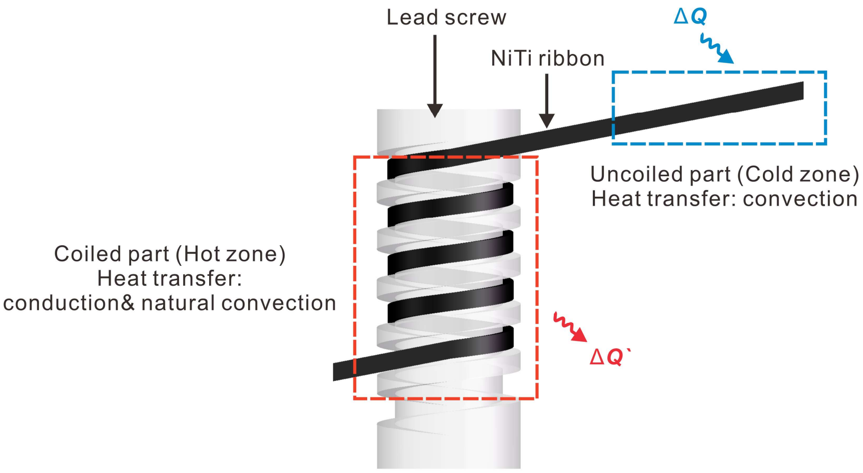

Fig. S12. The heat transfer of the NiTi ribbon/wire to the surrounding environment during the coiling-uncoiling cycle.

As shown in Fig. S12, the coiled hot NiTi ribbon/wire is in contact with the lead screw, which enables conduction heat losses. In contrast, the uncoiled cold NiTi ribbon/wire absorbs heat from the surrounding air though convection. The thermal conduction rate depends on the thermal conductivity of shape memory alloy (SMA) and the lead screw, the contact area, and the temperature gradient. As shown in Fig.S10, the characteristic heat transfer time (t_h) was 0.61 s for the NiTi wire in contact with the steel lead screw. In contrast, the t_h of the NiTi wire in contact with the resin lead screw reached 1.86 s, due to the low thermal conductivity of the resin. Thus, we used the resin lead screw to measure the adiabatic temperature change of NiTi during the coiling-uncoiling cycle. However, the conduction heat loss is still non-negligible for the resin lead screw. In addition, the contact area strongly affects the thermal conduction rate. The NiTi wire has a small diameter of 0.5 mm, and it is in line-contact with the lead screw. In contrast, the NiTi ribbon has a relatively large cross-sectional area of 1.5 × 0.5 mm^2, and it is in plane-contact with the lead screw. The conduction heat loss for the NiTi ribbon is much larger than that of the wire. Thus, the ΔT_{jump} (14.6 K) of the NiTi ribbon is lower than the ΔT_{drop}

(15.2 K). The ΔT_{jump} (14.7 K) of the NiTi wire is larger than the ΔT_{drop} (12.8 K), due to hysteresis heat and the low thermal conduction rate of line-contact.

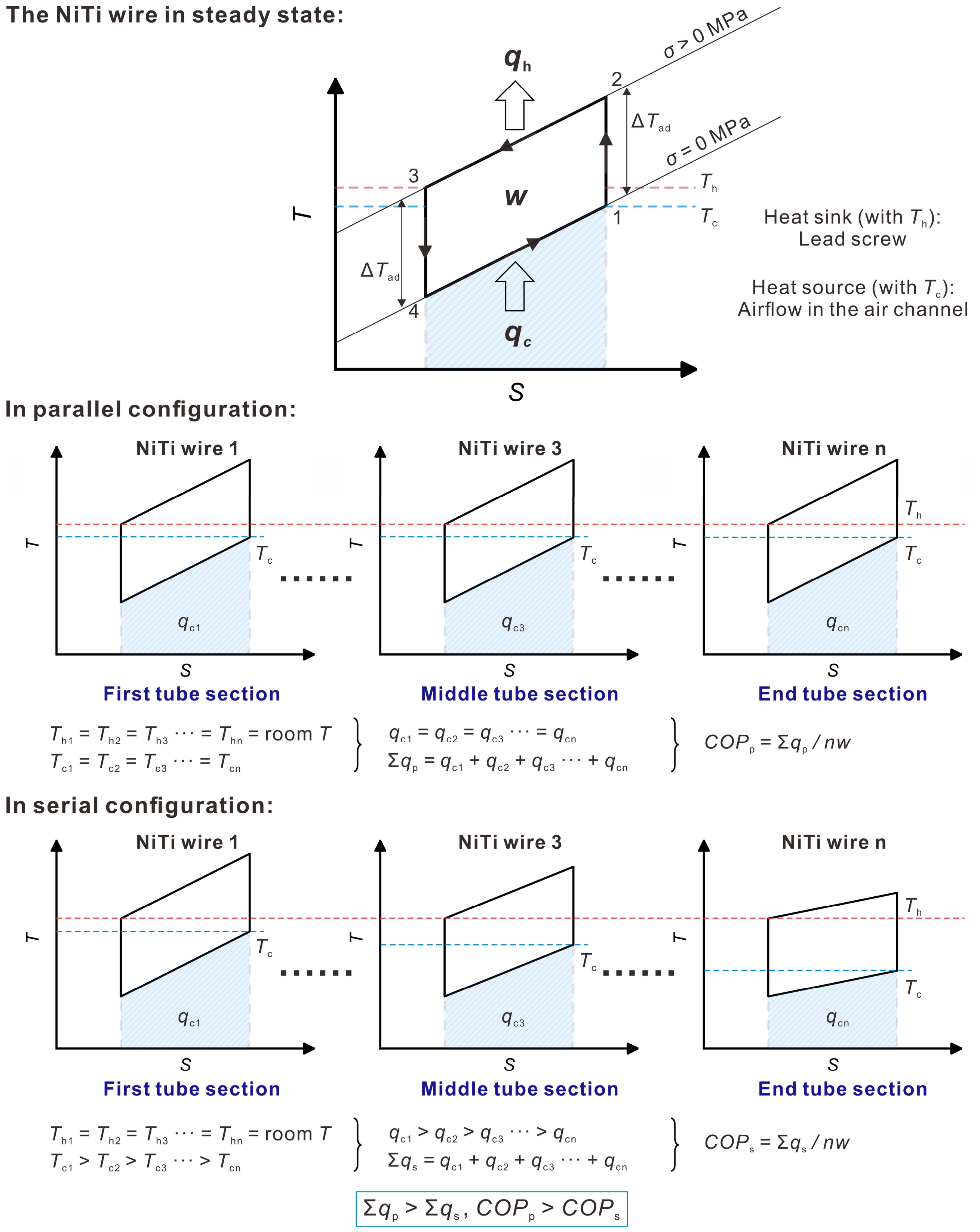

Fig. S13. Thermodynamic cycle of the NiTi wire in the coil-bending elastocaloric air cooler with different air

channel configurations.

As shown in Fig. S13, each single NiTi wire of the air cooler is considered as the subject of study in the thermodynamic system (steady state). The NiTi wire absorbed heat from the airflow and released heat to the lead screw. In this system, the heat sink is the lead screw with a temperature of T_h, while the heat source is the airflow in the silicon tube (as a control volume) with a temperature of T_c. The effect of heat accumulation in the lead screw was neglected for simplification. We assume that the material properties of the NiTi wires remain unchanged within the tested temperature range, allowing the adiabatic temperature change (ΔT_{ad}) and input work (w) to be treated as constants. The thermodynamic cycle, which is deduced inductively from the reverse Brayton cycle, starts with state 1 in austenite phase. The cycle includes four stages: adiabatic coiling (martensitic transformation) of the NiTi wire (state 1 to state 2), heat transfer from the wire to the lead screw (state 2 to state 3), adiabatic uncoiling (reverse transformation) of the NiTi wire (state 3 to state 4), heat transfer from the wire to the airflow (state 4 to state 1).The heat of the coiled NiTi wire is effectively transferred to the lead screw, leading the temperature of NiTi to drop to the T_h before uncoiling. The NiTi wire absorbs heat (q_c) from the heat source until its temperature reaches T_c. The NiTi wires in the parallel connection operate independently in each air tube section under the same heat transfer conditions. The convection heat transfer processes (state 2 to state 3, state 4 to state 1) are not linear in the reverse Brayton cycle; the straight lines are drawn in Fig. S13 for the purpose of simplification without affecting the thermodynamic analysis. In the steady state, the air cooler with parallel air channels exhibits a larger temperature difference between the wires and the airflow (less temperature difference between the T_h and the T_c), resulting in a large q_c and a large cooling power of the air cooler. In contrast, the serial

connection of the air channels enables a pre-cooling of the inlet airflow in the first few tube sections before entering the subsequent tube sections. As shown in the figure, the T_c decreases when the number of tube sections increases. The uncoiled NiTi wires in the following tube sections operate in a heat source with a lower T_c, leading to a lower q_c. The cooling power and coefficient of performance (*COP*) of the serial connection is lower than that of the parallel connection. The average temperature difference between airflow (T_c) and lead screw (T_h) is higher in the analysed sub-systems when using the serial configuration, which leads to a lower reversed Carnot cycle efficiency (T_c / (T_h −T_c)).

Note S7: Operation of the elastocaloric air cooler

Several working cycles are required when the elastocaloric air cooler operates from the initial state to the steady state. Generally, the required working cycles for the cooler stead state is related to the operating frequency and airflow rate. Here, one elastocaloric cooling cycle in our air cooler is comprised of four stages: forward motor rotation, holding for heat transfer between the airflow and NiTi wires, reverse motor rotation, and a second holding for the heat transfer. The test results of working cycle we considered are from the measured temperature of outlet airflow by the thermocouples. The measured results are summarized in the Tab. S2. As shown in the results, the required work cycles are increased with the operating frequency (f) and decreased with the air flowrate (ω). In the serial connected air channel, the required working cycles are decreased with the number of tube sections (n).

The heat accumulation in the lead screw caused the temperature rise of the lead screw. The average temperature rise of the lead screw (ΔT_h) increased and gradually stabilized during operation, where the largest ΔT_h was 3.1 K as shown in Fig. S14d. The ΔT_h increased with the air flow rate (ω) and the frequency (f). To mitigate the negative effect of heat accumulation on the cooling performance, one possible solution is to fabricate a hollow lead screw. By pumping heat transfer fluid (air) through the lead screw, the heat can be more quickly dissipated, which will further enhance the cooling power.

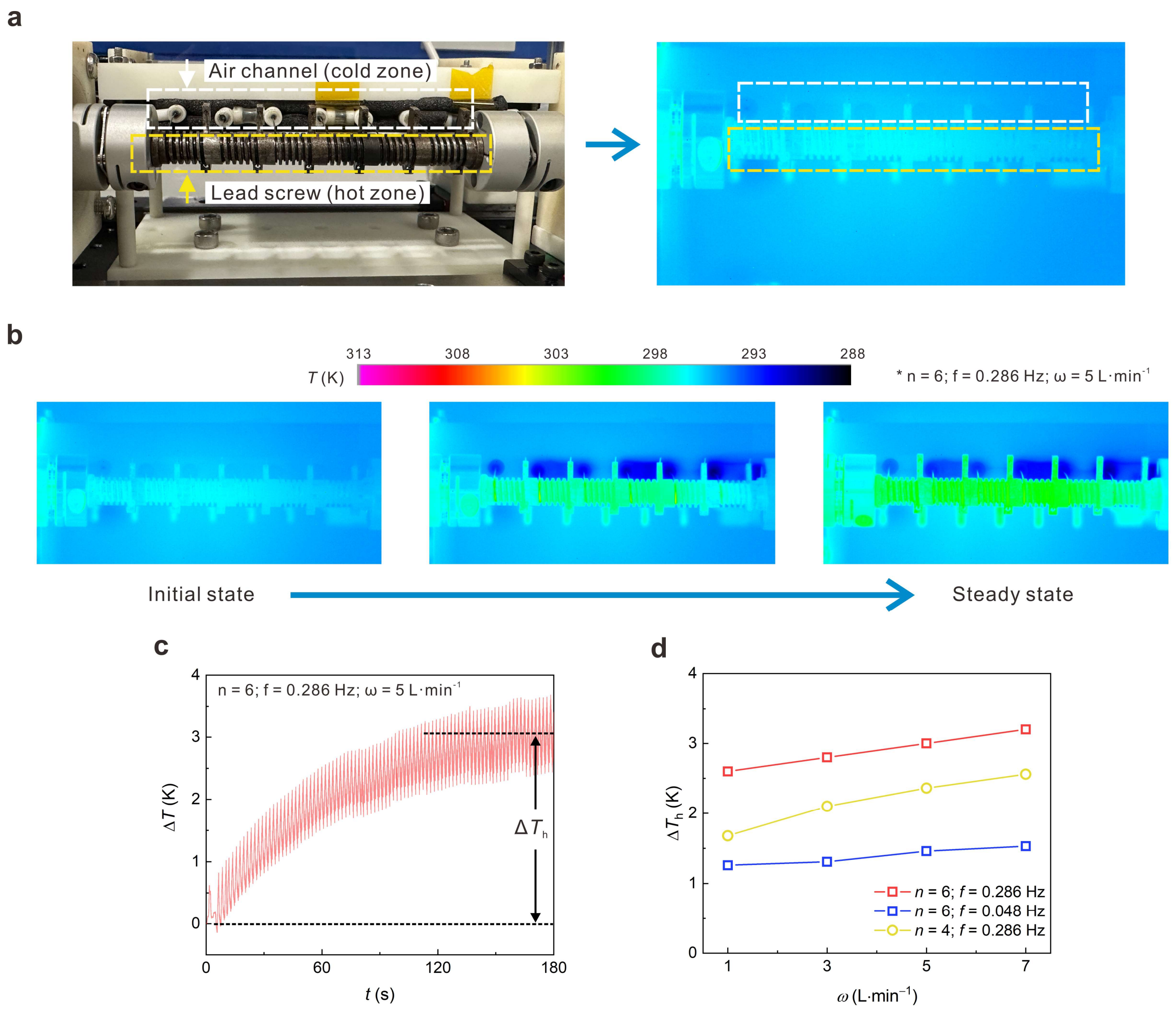

Fig. S14. a The elastocaloric air cooler and the corresponding thermographic image. **b** Thermographic images of the elastocaloric air cooler from the initial state to the steady state (at the operating conditions: n =6; f = 0.286 Hz; ω = 5 L·min^{-1}). **c** Temperature of the lead screw versus t (at the operating conditions: n =6; f = 0.286 Hz; ω = 5 L·min^{-1}). **d** ΔT_h versus ω under different operating conditions.

Tab. S2. Summary of the required working cycles from the initial state to the steady state of the cooler.

Air channel configuration	Operating conditions			
	ω	$n = 6; f = 0.286$ Hz	$n = 6; f = 0.048$ Hz	$n = 4; f = 0.286$ Hz
Serial connection	1 $L \cdot min^{-1}$	115	16	86
	3 $L \cdot min^{-1}$	65	13	50
	5 $L \cdot min^{-1}$	50	11	36
	7 $L \cdot min^{-1}$	30	8	26
	ω	$f = 0.286$ Hz	$f = 0.048$ Hz	
Parallel connection	1 $L \cdot min^{-1}$	68	10	
	3 $L \cdot min^{-1}$	28	8	
	5 $L \cdot min^{-1}$	20	7	

Note S8: Torque measurement of coil-bending

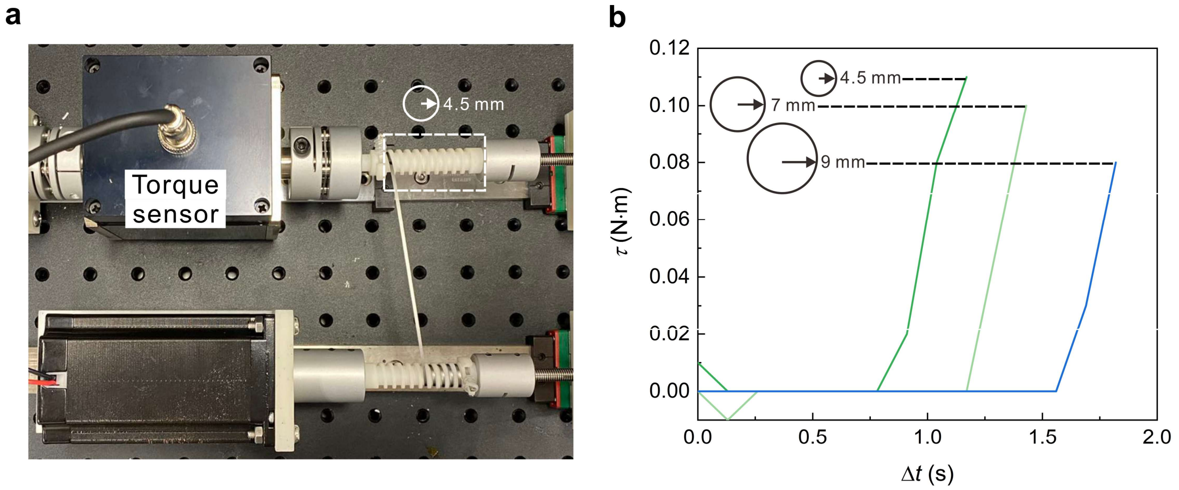

Fig. S15. a The experimental setup. **b** Torques applied on a NiTi ribbon in adiabatic coil-bending.

The torque sensor (JN-DN1) was installed along the axis direction of the shaft. The torque depended on the radius of the lead screw and the mass of the coiled NiTi ribbon. As shown in Fig. S15a, we recorded the required torque for one adiabatic coiling-uncoiling process with a rotation duration of 0.5 seconds. The number of rotations of the motors was adjusted to ensure that the same mass of the NiTi ribbon was coiled on the lead screw as the radius changed. The measured results are summarized in the Tab. S3. The recorded torque is presented in Fig. S15b, and we used the maximum value to calculate the required force. The specific driving force F_{sp} is calculated by Eq. (s16).

$$F_{sp} = \frac{\Delta\tau}{r \cdot m_{NiTi}} \qquad \text{(s16)}$$

Tab. S3. Summary of the testing parameters and measured results in adiabatic condition.

	Type 1	Type 2	Type 3
r	4.5 mm	7 mm	9.5 mm
r_c	4.84 mm	7.31 mm	9.79 mm
$\varepsilon_{\text{max, pure}}$	5.17%	3.42%	2.55%
$\varepsilon_{\text{max, shift}}$	7.01%	4.25%	2.88%
Rotated turns	4	2.7	2
m_{NiTi} on lead screw	0.588 g	0.598 g	0.595 g
$\Delta\tau$	0.11 N·m	0.0975 N·m	0.088 N·m
F_{sp}	41.6 $\text{N}\cdot\text{g}^{-1}$	23.2 $\text{N}\cdot\text{g}^{-1}$	15.6 $\text{N}\cdot\text{g}^{-1}$

The experimental setup for the torque measurement in the elastocaloric air cooler is shown in Fig. S16a. Due to the symmetrical structure, the NiTi wires were only coiled on one lead screw, with one motor using mechanical work to drive the NiTi wires in each rotation. The average $\Delta\tau$ for two motors was the same absolute value (rotation direction is different), as shown schematically in Fig. S15b. Six NiTi wires were used in the test at an operational frequency of 0.143 Hz (the middle value of operational frequencies in text). Each cycle consisted of forward rotation for 2.5 s, holding for 1 s, reverse rotation for 2.5 s, and second holding for 1 s. The recorded torque of the motor versus time is shown in Fig. S17, showing the periodic pattern. The average specific required torque $\overline{\Delta\tau_{\text{sp}}}$ is calculated by Eq. (s17), where the integration of $\Delta\tau$ (the area below the baseline) is calculated; Δt is duration of the torque applied on the shaft, including two rotation duration and one holding duration (here for 6 s); $m_{\text{NiTi, flow}}$ is the total mass of NiTi wires in the air channels, equal to the mass of coiled NiTi wires on the lead screw.

$$\overline{\Delta\tau_{\text{sp}}} = \frac{\int \Delta\tau dt}{\Delta t \cdot m_{\text{NiTi,flow}}} \quad \text{(s17)}$$

The peak value of $\Delta\tau$ shown in Fig. S11 is 0.07 N·m. Thus, the specific driving force of the elastocaloric air cooler F_{sp} is 26 N·g^{-1} calculated by Eq. (s16).

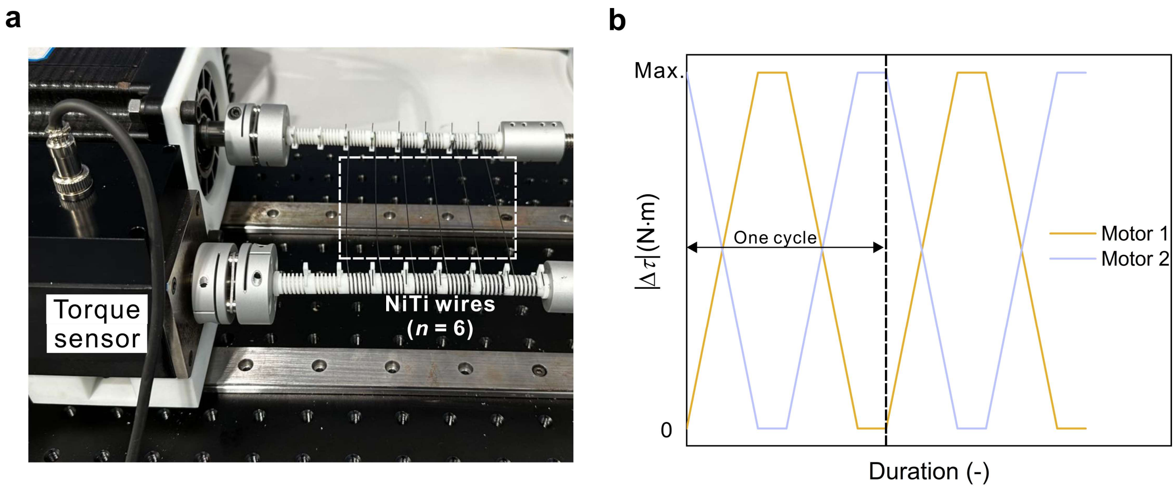

Fig. S16. **a** The experimental setup for torque measurement. **b** Schematic absolute torque changes of the elastocaloric air cooler during operation.

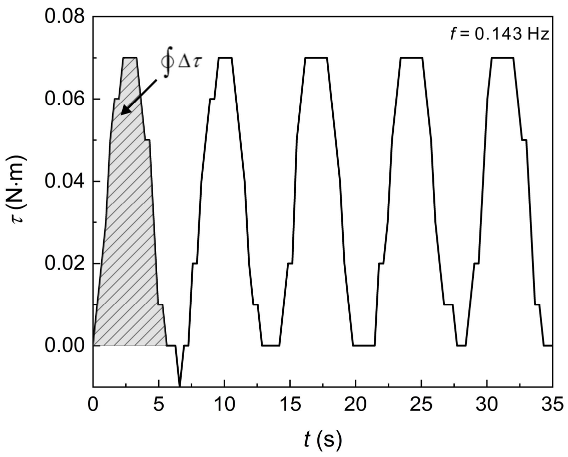

Fig. S17. Recorded torque outputted by one motor at $f = 0.143$ Hz.

Note S9: Mechanical input power of the elastocaloric air cooler

The mechanical input power in the elastocaloric air cooler is generated by the motors. Therefore, we calculated the mechanical input power by multiplying the torque with the angular velocity of the motors. In the operation of the elastocaloric air cooler, we tested four different operational frequencies with the same number of rotations (5 turns in forward or reverse rotation). It should be noted that only one motor inputs mechanical work to the system in each rotation process (forward or reverse rotation). The power of the air pump is very small compared with the mechanical work from the motor and thus was not considered in the *COP* calculation[6]. The specific mechanical input power of elastocaloric air cooler $\dot{w}_{\mathrm{mech}}$ is calculated by Eq. (s18),

$$\dot{w}_{\mathrm{mech}} = \overline{\Delta \tau_{\mathrm{sp}}} \cdot 2\pi \cdot n_{\mathrm{rps}} \cdot \frac{P_{\mathrm{r}}}{P} \tag{s18}$$

where n_{rps} is the number of rotated turns per second and angular velocity is equal to $2\pi n_{rps}$; P_{r} is the duration of rotation including the forward and reverse rotations; P is the total duration of one elastocaloric cooling cycle; $\overline{\Delta\tau_{\mathrm{sp}}}$ is the average specific torque change. The summary is shown in Tab. S4.

Tab. S4. Summary of operational conditions of the elastocaloric air cooler.

	$f = 0.048$ Hz	$f = 0.071$ Hz	$f = 0.143$ Hz	$f = 0.286$ Hz
P	21 s	14 s	7 s	3.5 s
P_r	15 s	10 s	5 s	2.5 s
Rotated turns	5 (each rotation process)			
n_{rps}	0.67 s^{-1}	1 s^{-1}	2 s^{-1}	4 s^{-1}
r	4.5 mm			
$\overline{\Delta\tau_{\text{sp}}}$	0.0683 $N \cdot m \cdot g^{-1}$			
F_{sp}	26 $N \cdot g^{-1}$			
$\dot{w}_{\text{mech}}$	0.205 $W \cdot g^{-1}$	0.306 $W \cdot g^{-1}$	0.612 $W \cdot g^{-1}$	1.224 $W \cdot g^{-1}$

Tab. S5. Fatigue life of the used NiTi wires under different strain levels.

	Type 1	Type 2	Type 3
r	4.5 mm	7 mm	9.5 mm
$\varepsilon_{\max}$	~ 7.0%	~ 4.2%	~ 2.8%
Number of cycles	3317	6537	10723

Note S10: Overview of the state-of-the-art elastocaloric cooling prototypes

Tab. S6. Performance summary of state-of-the-art elastocaloric cooling prototypes and demos.

Actuation	ΔT span (K)	q (W)	F_{sp} ($N \cdot g^{-1}$)	Material (-)	Heat transfer (-)	Reference
Compression	1.5	38	630	NiTi tube	Water convection	7
	5	11.2	10227			8
	5.6	7.9	16666			9
	31.3	48.7	2200			10
	22.5	260	630			11
	5.7	4	380			12
	9.1	3.4	380			13
	0.8	/	/		Air convection	14
	4.7	65	630		Water convection	15
	27.7	6	2480	NiTi section topology		16
	8	4.2	2469			17
	50.6	203	240			18
Tension	9.2	3.1	385	NiTi wire	Air convection	19
	30 (simulated)	1000 (simulated)	/	NiMnTiB/NiTiCuV wire		20
	28.5 (simulated)	1240 (simulated)	/	NiTiCuV wire		21
	2.5	/	/	NiTi/NiTiCuCo wire		22
	1.5	/	/			23
	28.3	1.3	315	NiTi wire	Water convection	24
	15.3	4.5 (heat pump)	1250	NiTi sheet		6
	19.9	/	1250			26
	5	0.9	/		Solid-solid conduction	27
	14	0.2	/			28
Bending	6	/	/			29
	6	1500 (simulated)	/	NiTi wire		30
	23.8 (simulated)	60 (simulated)	/		Air convection	31
	5.5	11.5	5.7	NiTi sheet		2
Torsion	12	/	/	NiTi wire	Water convection	25

References

1. Wick, A., Vöhringer, O. & Pelton, A. R. The Bending Behavior of NiTi. *Journal de Physique IV* **05**, C8-789-C8-794 (1995). https://doi.org/10.1051/jp4/199558789
2. Li, X., Cheng, S. & Sun, Q. A compact NiTi elastocaloric air cooler with low force bending actuation. *Appl. Therm. Eng.* **215**, 118942 (2022). https://doi.org/10.1016/j.applthermaleng.2022.118942
3. Reedlunn, B. et al. Tension, compression, and bending of superelastic shape memory alloy tubes. *J. Mech. Phys. Solids.* **63**, 506–537 (2014). https://doi.org/10.1016/j.jmps.2012.12.012
4. JCGM. Evaluation of measurement data-Guide to the expression of uncertainty in measurement. *Int. Organ. Stand. Geneva* ISBN **50** (2008).
5. He, Y., Yin, H., Zhou, R. & Sun, Q. Ambient effect on damping peak of NiTi shape memory alloy. *Mater. Lett.* **64**, 1483–1486 (2010). https://doi.org/10.1016/j.matlet.2010.03.068
6. Tušek, J. et al. A regenerative elastocaloric heat pump. *Nat. Energy* **1**, 1–6 (2016). https://doi.org/10.1038/nenergy.2016.134
7. Qian, S. et al. Experimental Evaluation of a Compressive Elastocaloric Cooling System. *16th International Refrigeration and Air Conditioning Conference at Purdue* **16**, 2385 (2016). http://docs.lib.purdue.edu/iracc/1726
8. Ianniciello, L., Bartholomé, K., Fitger, A. & Engelbrecht, K. Long life elastocaloric regenerator operating under compression. *Appl. Therm. Eng.* **202**, 117838 (2022). https://doi.org/10.1016/j.applthermaleng.2021.117838
9. Bachmann, N. et al. Long-term stable compressive elastocaloric cooling system with latent heat transfer. *Commun. Phys.* **4**, 1–6 (2021). https://doi.org/10.1038/s42005-021-00697-y
10. Ahčin, Ž. et al. High-performance cooling and heat pumping based on fatigue-resistant elastocaloric effect in compression. *Joule* **6**, 2338–2357 (2022). https://doi.org/10.1016/j.joule.2022.08.011
11. Qian, S. et al. High-performance multimode elastocaloric cooling system. *Science* **380**, 722–727 (2023). https://doi.org/10.1126/science.adg7043
12. Cheng, S. et al. Buckling prevention of a single long NiTi tube compressive elastocaloric regenerator. I*nt. J. Solids Struct.* **271–272**, 112263 (2023). https://doi.org/10.1016/j.ijsolstr.2023.112263
13. Cheng, S. et al. Development of a crankshaft driven single long NiTi tube compressive elastocaloric cooler. *Sci. Technol. Built. Environ.*, 1–14 In Press (2023). https://doi.org/10.1080/23744731.2023.2242756
14. Jongchansitto, P., Yachai, T., Preechawuttipong, I., Boufayed, R. & Balandraud, X. Concept of mechanocaloric granular material made from shape memory alloy. *Energy* **219**, 119656 (2021). https://doi.org/10.1016/j.energy.2020.119656
15. Qian, S. et al. Design of a hydraulically driven compressive elastocaloric cooling system. *Sci. Technol. Built. Environ.* **22**, 500–506 (2016). https://doi.org/10.1080/23744731.2016.1171630
16. Zhang, J., Zhu, Y., Yao, S. & Sun, Q. Highly Efficient Grooved NiTi Tube Refrigerants for Compressive Elastocaloric Cooling. *Appl. Therm. Eng.* **228**, 120439 (2023) https://doi.org/10.1016/j.applthermaleng.2023.120439
17. Zhang, J., Zhu, Y., Cheng, S., Yao, S. & Sun, Q. Enhancing cooling performance of NiTi elastocaloric tube refrigerant via internal grooving. *Appl. Therm. Eng.* **213**, 118657 (2022). https://doi.org/10.1016/j.applthermaleng.2022.118657
18. Zhou, G., Zhu, Y., Yao, S. & Sun, Q. Giant temperature span and cooling power in elastocaloric regenerator. *Joule* **7**, 2003-2015 (2023). https://doi.org/10.1016/j.joule.2023.07.004
19. Chen, Y., Wang, Y., Sun, W., Qian, S. & Liu, J. A compact elastocaloric refrigerator. *The Innovation* **3**,

100205 (2022). https://doi.org/10.1016/j.xinn.2022.100205
20. Borzacchiello, A., Cirillo, L., Greco, A. & Masselli, C. A comparison between different materials with elastocaloric effect for a rotary cooling prototype. *Appl. Therm. Eng.* **235**, 121344 (2023). https://doi.org/10.1016/j.applthermaleng.2023.121344
21. Cirillo, L., Greco, A. & Masselli, C. The energy performances of an elastocaloric device for air conditioning through numerical investigation. *Appl. Therm. Eng.* 121517, In Press (2023). https://doi.org/10.1016/j.applthermaleng.2023.121517
22. Kirsch, S. M. et al. Continuous operating elastocaloric heating and cooling device: Air flow investigation and experimental parameter study. *SMASIS2019*. (2019)
23. Michaelis, N. et al. Investigation of Elastocaloric Air Cooling Potential Based on Superelastic SMA Wire Bundles. *SMASIS20* (2020).
24. Snodgrass, R. & Erickson, D. A multistage elastocaloric refrigerator and heat pump with 28 K temperature span. *Sci. Rep.* **9**, 1–10 (2019). https://doi.org/10.1038/s41598-019-54411-8
25. Wang, R. et al. Torsional refrigeration by twisted, coiled, and supercoiled fibers. *Science* **366**, 216–221 (2019). https://doi.org/10.1126/science.aax6182
26. Engelbrecht, K. et al. A regenerative elastocaloric device: experimental results. *J. Phys. D: Appl. Phys.* **50**, 424006 (2017). https://doi.org/10.1088/1361-6463/aa8656
27. Ulpiani, G. et al. Upscaling of SMA film-based elastocaloric cooling. *Appl. Therm. Eng.* **180**, 115867 (2020). https://doi.org/10.1016/j.applthermaleng.2020.115867
28. Bruederlin, F. et al. Elastocaloric Cooling on the Miniature Scale: A Review on Materials and Device Engineering. *Energy Technol.* **6**, 1588–1604 (2018). https://doi.org/10.1002/ente.201800137
29. Cheng, S. et al. Continuous rotating bending NiTi sheets for elastocaloric cooling: Model and experiments. *Int. J. Refrig.* **147**, 39–47 (2023). https://doi.org/10.1016/j.ijrefrig.2022.11.020
30. Sharar, D. J., Radice, J., Warzoha, R., Hanrahan, B. & Smith, A. Low-force elastocaloric refrigeration via bending. *Appl. Phys. Lett.* **118**, 184103 (2021). https://doi.org/10.1063/5.0041500
31. Masselli, C., Cirillo, L. & Greco, A. Cooling of electronic circuits through elastocaloric solid-state technology: A numerical analysis for the development of the CHECK TEMPERATURE prototype. *Appl. Therm. Eng.* **230**, 120729 (2023). https://doi.org/10.1016/j.applthermaleng.2023.121344
