# Supplementary material for: Continuous and efficient elastocaloric air cooling by coil-bending
Source: Nat Commun. 2023 Dec 2;14:7982. doi: 10.1038/s41467-023-43611-6 (PMC10693641; doi:10.1038/s41467-023-43611-6)
Supplement: Supplementary file 3 — Description of Additional Supplementary Files [file 41467_2023_43611_MOESM3_ESM.pdf]

Description of Additional Supplementary Files

File Name: Supplementary Movie 1
Description: Demonstration of coil-bending and elastocaloric air cooling prototype
